# Network medicine framework for identifying drug-repurposing opportunities for COVID-19

**DOI:** 10.1073/pnas.2025581118

**Published:** 2021-04-27

**Authors:** Deisy Morselli Gysi, Ítalo do Valle, Marinka Zitnik, Asher Ameli, Xiao Gan, Onur Varol, Susan Dina Ghiassian, J. J. Patten, Robert A. Davey, Joseph Loscalzo, Albert-László Barabási

**Affiliations:** ^a^Network Science Institute, Northeastern University, Boston, MA 02115;; ^b^Department of Physics, Northeastern University, Boston, MA 02115;; ^c^Channing Division of Network Medicine, Department of Medicine, Brigham and Women’s Hospital, Harvard Medical School, Boston, MA 02115;; ^d^Department of Biomedical Informatics, Harvard University, Boston, MA 02115;; ^e^Harvard Data Science Initiative, Harvard University, Cambridge, MA 02138;; ^f^Data Science Department, Scipher Medicine, Waltham, MA 02453;; ^g^Faculty of Engineering and Natural Sciences, Sabanci University, Istanbul 34956, Turkey;; ^h^Department of Microbiology, National Emerging Infectious Diseases Laboratories, Boston University, Boston, MA 02118;; ^i^Department of Medicine, Brigham and Women’s Hospital, Harvard Medical School, Boston, MA 02115;; ^j^Department of Network and Data Science, Central European University, Budapest 1051, Hungary

**Keywords:** systems biology, network medicine, drug repurposing, infectious diseases

## Abstract

The COVID-19 pandemic has highlighted the need to quickly and reliably prioritize clinically approved compounds for their potential effectiveness for severe acute respiratory syndrome coronavirus 2 (SARS-CoV-2) infections. Here, we deployed algorithms relying on artificial intelligence, network diffusion, and network proximity, tasking each of them to rank 6,340 drugs for their expected efficacy against SARS-CoV-2. To test the predictions, we used as ground truth 918 drugs experimentally screened in VeroE6 cells, as well as the list of drugs in clinical trials that capture the medical community’s assessment of drugs with potential COVID-19 efficacy. We find that no single predictive algorithm offers consistently reliable outcomes across all datasets and metrics. This outcome prompted us to develop a multimodal technology that fuses the predictions of all algorithms, finding that a consensus among the different predictive methods consistently exceeds the performance of the best individual pipelines. We screened in human cells the top-ranked drugs, obtaining a 62% success rate, in contrast to the 0.8% hit rate of nonguided screenings. Of the six drugs that reduced viral infection, four could be directly repurposed to treat COVID-19, proposing novel treatments for COVID-19. We also found that 76 of the 77 drugs that successfully reduced viral infection do not bind the proteins targeted by SARS-CoV-2, indicating that these network drugs rely on network-based mechanisms that cannot be identified using docking-based strategies. These advances offer a methodological pathway to identify repurposable drugs for future pathogens and neglected diseases underserved by the costs and extended timeline of de novo drug development.

## Significance

The COVID-19 pandemic has highlighted the importance of prioritizing approved drugs to treat severe acute respiratory syndrome coronavirus 2 (SARS-CoV-2) infections. Here, we deployed algorithms relying on artificial intelligence, network diffusion, and network proximity to rank 6,340 drugs for their expected efficacy against SARS-CoV-2. We experimentally screened 918 drugs, allowing us to evaluate the performance of the existing drug-repurposing methodologies, and used a consensus algorithm to increase the accuracy of the predictions. Finally, we screened in human cells the top-ranked drugs, identifying six drugs that reduced viral infection, four of which could be repurposed to treat COVID-19. The developed strategy has significance beyond COVID-19, allowing us to identify drug-repurposing candidates for neglected diseases.

The disruptive nature of the COVID-19 pandemic has unveiled the need for the rapid development, testing, and deployment of new drugs and cures. Given the compressed timescales, the de novo drug development process, which typically lasts a decade or longer, is not feasible. A time-efficient strategy must rely on drug repurposing (or repositioning), helping identify among the compounds approved for clinical use the few that may also have a therapeutic effect in patients with COVID-19. Yet, the lack of reliable repurposing methodologies has resulted in a winner-takes-all pattern, where more than one-third of registered clinical trials focus on hydroxychloroquine or chloroquine, siphoning away resources from testing a wider range of potentially effective drug candidates. While a full unbiased screening of all approved drugs could identify all possible treatments, given the combination of its high cost, extended timeline, and exceptionally low success rate (1), we need efficient strategies that enable effective drug prioritization.

Drug-repurposing algorithms rank drugs based on one or multiple streams of information, such as molecular profiles (2), chemical structures (3), adverse profiles (4), molecular docking (5), electronic health records (6), pathway analysis (7), genome wide association studies (7), and network perturbations (7–15). Yet, typically only a small subset of the top candidates is validated experimentally; hence, the true predictive power of the existing repurposing algorithms remains unknown. To quantify and compare their true predictive power, all algorithms must make predictions for the same set of candidates, and the experimental validation must focus not only on the top candidates, as it does now, but on a wider list of drugs chosen independently of their predicted rank.

**Fig. 1.**
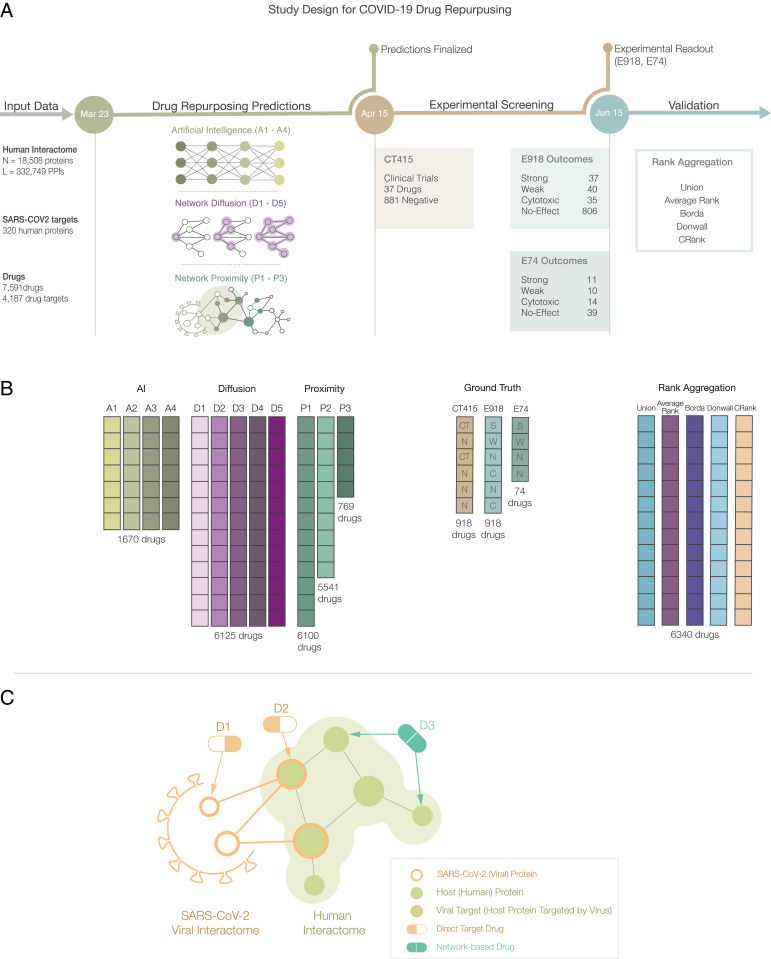
Network medicine framework for drug repurposing. (*A*) Study design and timeline. Following the publication of host–pathogen PPIs (21) (March 23, 2020), we implemented three drug-repurposing algorithms, relying on AI (A1 to A4), network diffusion (D1 to D5), and proximity (P1 to P3), together resulting in 12 predictive ranking lists (pipelines, shown in *B*). Each pipeline offers predictions for a different number of drugs that were frozen on April 15, 2020. We then identified 918 drugs for which all pipelines but P3 offered predictions, and experimentally validated their effect on the virus in VeroE6 cells (18). The experimental (E918, E74) and clinical trial lists C415 offered the ground truth for validation and rank aggregation. (*C*) Direct target drugs bind either to a viral protein (D1) or to a host protein target of the viral proteins (D2). Network drugs (D3), in contrast, bind to the host proteins and limit viral activity by perturbing the host subcellular network.

The COVID-19 pandemic presents both the societal imperative and the rationale to test drugs at a previously unseen scale. Hence, it offers a unique opportunity to quantify and improve the efficacy of the available predictive algorithms, while also identifying potential treatments for COVID-19. Here, we implement three network-medicine drug-repurposing algorithms that rely on artificial intelligence (AI) (15, 16), network diffusion (17), and network proximity (11) (Fig. 1 *A* and *B*). To test the validity of the predictions, we identified 918 drugs ranked by all predictive pipelines, and experimentally screened them to identify those that inhibit viral infection and replication in cultured nonhuman primate cells (18); the successful outcomes were further validated in human-derived cells. We also collected clinical trial data to capture the medical community’s collective assessment of drug candidates. We found that the predictive power varies for the different datasets and metrics, indicating that in the absence of a priori ground truth, it is impossible to determine which algorithm to trust. Our key advance, therefore, is a multimodal ensemble forecasting approach that significantly improves the accuracy and the reliability of the predictions by seeking consensus among the predictive methods (15, 19).

## Results

### Network-Based Drug Repurposing.

**Fig. 2.**
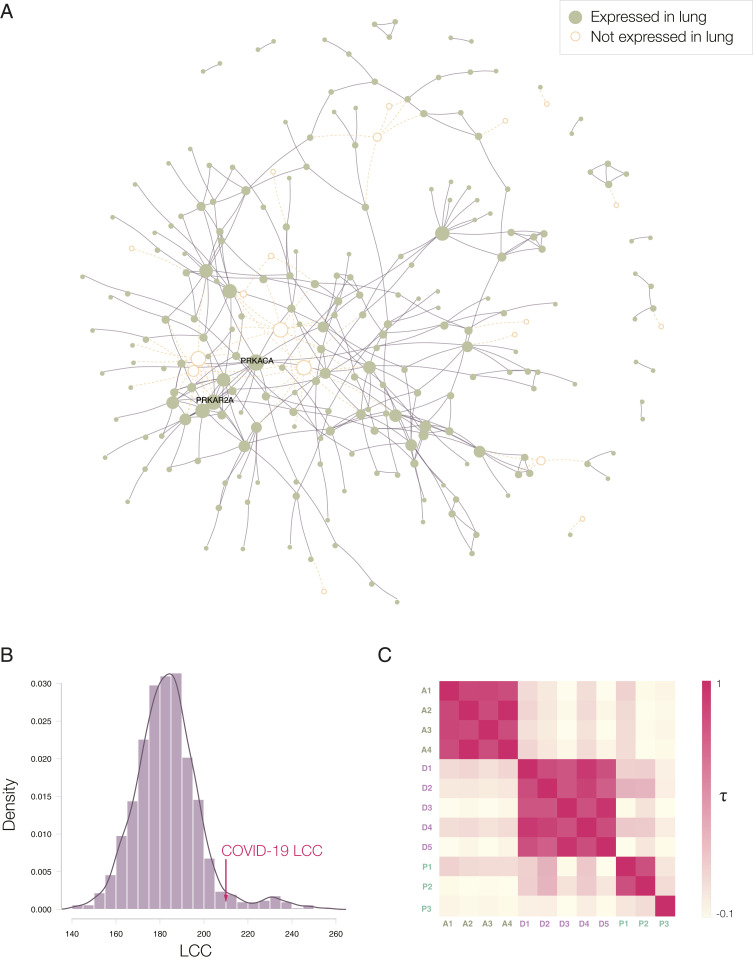
COVID-19 disease module. (*A*) Proteins targeted by SARS-CoV-2 are not distributed randomly in the human interactome, but form a large connected component (LCC) consisting of 208 proteins, and multiple small subgraphs, shown in the figure. Almost all proteins in SARS-CoV-2 LCC are also expressed in the lung tissue, potentially explaining the effectiveness of the virus in causing pulmonary manifestations of the disease. (*B*) The random expectation of the LCC size indicates that the observed COVID-19 LCC, whose size is indicated by the red arrow, is larger than expected by chance (*z*-score = 1.65). (*C*) Heatmap of the Kendall τ statistic showing that the ranking list predicted by the different methods (A, D, and P) are not correlated. We observe, however, high correlations among the individual ranking list predicted by the same predictive method.

Repurposing strategies often prioritize drugs approved for (other) diseases whose molecular manifestations are similar to those caused by the pathogen or disease of interest (20). To search for diseases whose molecular mechanisms overlap with the COVID-19 disease, we first mapped the experimentally identified (21) 332 host protein targets of the severe acute respiratory syndrome coronavirus 2 (SARS-CoV-2) proteins (Dataset S1) to the human interactome (22–25) (Dataset S2), a collection of 332,749 pairwise binding interactions between 18,508 human proteins (*SI Appendix*, Section 1.1). We found that 208 of the 332 viral targets form a large connected component (hereafter denoted the COVID-19 disease module) (Fig. 2*B*), indicating that the SARS-CoV-2 targets aggregate in the same network vicinity (13, 20). Next, we evaluated the network-based overlap between proteins associated with 299 diseases (26) (*d*) and the host protein targets of SARS-CoV-2 (*v*) using the *S*_*vd*_ metric (26), finding *S*_*vd*_ > 0 for all diseases, implying that the COVID-19 disease module does not directly overlap with the disease proteins associated with any single disease (*SI Appendix*, Figs. S1 and S2 and Dataset S5). In other words, a potential COVID-19 treatment cannot be derived from the arsenal of therapies approved for a specific disease, arguing for a network-based strategy that can identify repurposable drugs without regard for their established disease indication.

We implemented three competing network-repurposing methodologies (Fig. 1*B* and *SI Appendix*, Section 2). 1) The AI-based algorithm (15, 16) maps drug protein targets and disease-associated proteins to points in a low-dimensional vector space, resulting in four predictive pipelines (A1 to A4) that rely on different drug-disease embeddings. 2) The diffusion algorithm (17) is inspired by diffusion state distance, and ranks drugs based on capturing network similarity of a drug’s protein targets to the SARS-CoV-2 host protein targets. Powered by distinct statistical measures, the algorithm offers five ranking pipelines (D1 to D5). 3) The proximity algorithm (11) ranks drugs based on the distance between the host protein targets of SARS-CoV2 and the closest protein targets of drugs, resulting in three predictive pipelines, of which: P1 relies on all drug targets; P2 tests the hypothesis that removing the protein targets involved in drug delivery and drug metabolism, shared by multiple drugs, can improve the specificity of the proximity measure; and P3 (Dataset S4) tests if drug-induced differentially expressed genes can offer additional predictive power (27). The low correlations across the three algorithms indicate that the methods extract complementary information from the network (Fig. 2*C* and *SI Appendix*, Section 3.2).

### Experimental and Clinical Validation of Drug-Repurposing Pipelines.

We implemented the 12 pipelines to predict the expected efficacy of 6,340 drugs in Drugbank (27) against SARS-CoV-2, and extracted and froze the predictions in the form of 12 ranked lists on April 15, 2020. All pipelines rely on the same input data and, to maintain the prospective nature of the study, all subsequent analyses rely on this initial prediction list. As the different pipelines make successful predictions of a different subset of drugs, we identified 918 drugs for which all pipelines (except for P3, which predicts the smallest number of drugs) offer predictions and whose compounds were available in the Broad Institute drug-repurposing library (28) (Fig. 1); we used two independent datasets to quantify the predictive power of each pipeline over the same set of drugs.

**Fig. 3.**
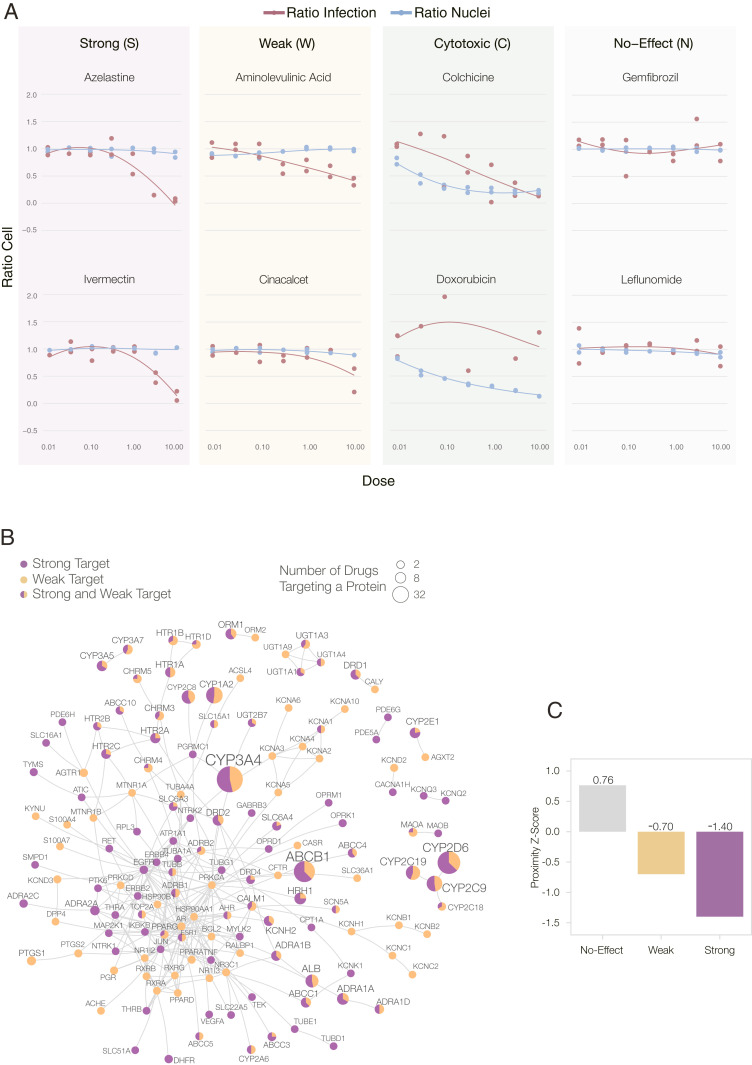
Experimental outcomes and network origins. (*A*) Examples of dose–response curves for eight of the 918 experimentally validated drugs (18), illustrating the four observed outcomes (S, W, C, and N). VeroE6 cells were challenged in vitro with SARS-CoV-2 virus and treated with the drug over a range of doses (from 8 nM to 8 µM). A two-step drug-response model (*SI Appendix*, Section 4.3) was used to classify each drug into S, W, C, or N categories, according to their response to the drug in different doses and cell and viral reduction. (*B*) The subnetwork formed by the targets of the 77 S&W drugs within the interactome. The link corresponds to binding interactions. Purple proteins are targeted by S drugs only; orange by W drugs only; proteins targeted by both S&W drugs are shown as pie charts, proportional to the number of targets in each category. (*C*) The targets of N drugs have a positive proximity *z*-score to the COVID-19 module, meaning they are further from the COVID-19 module than random expectation. In contrast, the targets of S&W drugs are more proximal (closer) to the COVID-19 module than expected by change, suggesting that their COVID-19 vicinity contribute to their ability to alter the virus’s ability to infect the cells.

As the first ground truth, we compare our predictions against the 918 compounds that had been experimentally screened for their efficacy against SARS-CoV-2 in VeroE6 cells, kidney epithelial cells derived from African green monkey (18) (*SI Appendix*, Section 4), experiments performed after the predictions were finalized (Fig. 1*A*). Briefly, the VeroE6 cells were preincubated with the drugs (from 8 µM down to 8 nM) and then challenged with wild-type SARS-CoV-2 strain USA-WA1/2020. Of the 918 drugs, 806 had no detectable effect on viral infectivity (N drugs, 87.8% of the tested list); 35 were cytotoxic to the host cells (C drugs); 37 had a strong effect (S drugs), being active over a broad range of concentrations; and 40 had a weak effect (W drugs) on the virus (Fig. 3*A* and Dataset S8). As the prediction pipelines offer no guidance on the magnitude of the in vivo effect, we considered as positive outcomes drugs that had a strong or a weak effect on the virus (S&W, 77 drugs) (Table 1), and as negative outcomes the drugs without detectable effect (N, 806 drugs).

**Table 1.** Drugs with positive experimental outcomes

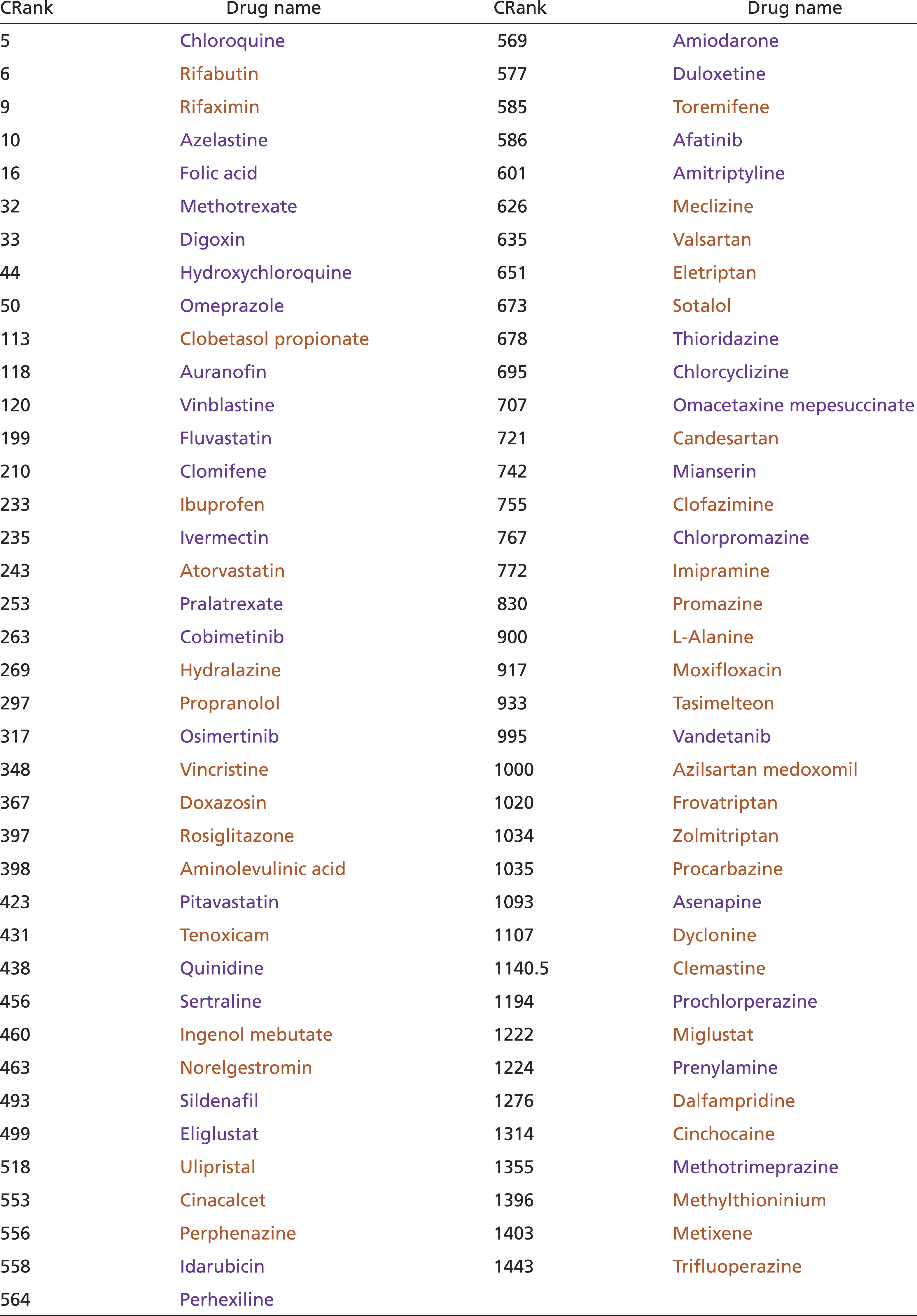

List of the 77 drugs with a positive outcome (S&W) from in vitro screen (18). Drug response classification was obtained by a two-step model for drug response (*SI Appendix*, Section 4.3). Drugs in purple show strong effect (S), and in orange show weak effect (W).

Second, on April 15, 2020 (prediction date), we scanned clinicaltrials.gov, identifying 67 drugs in 134 clinical trials for COVID-19 (CT415 dataset) (Dataset S10). To compare outcomes across datasets, we limited our analysis to the experimentally tested 918 drugs, considering as positive the 37 drugs in clinical trial on the E918 list, and as negative the remaining 881 drugs. As the outcomes of these trials are largely unknown, validation against the CT415 dataset tests each pipeline’s ability to predict the pharmacological consensus of the medical community on drugs with expected potential efficacy for COVID-19 patients.

**Fig. 4.**
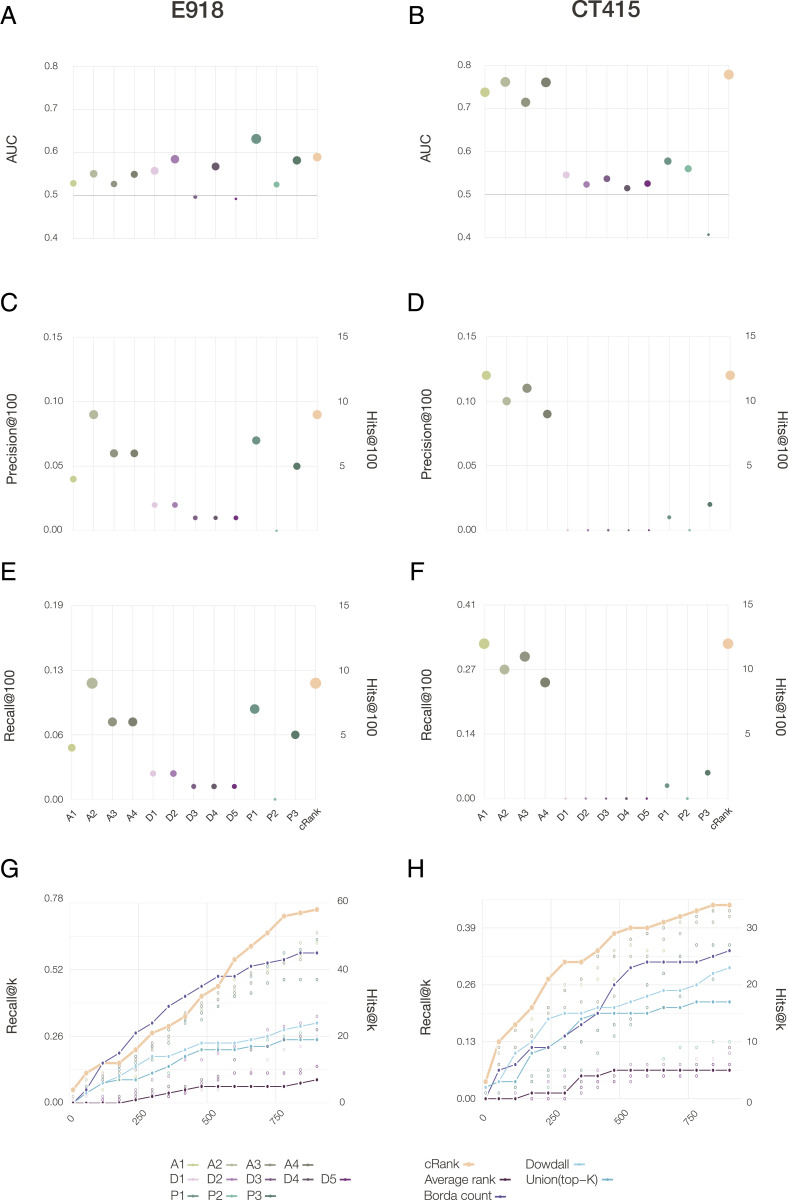
Performance of the predictive pipelines. (*A* and *B*) AUC, (*C* and *D*) precision at 100, and (*E* and *F*) recall at 100, for 12 pipelines tested for drug repurposing, each plot using as a gold standard the S&W drugs in E918 (left column) and drugs under clinical trials for treating COVID-19 as of April 15th, 2020 (CT415, right column). (*G* and *H*) The top *K* precision and recall for the different rank aggregation methods (connected points), compared to the individual pipelines (empty symbols) documenting the consistent predictive performance of CRank. Similar results are shown for two other datasets in *SI Appendix*, Fig. S8: the prospective expert curated E74 and the clinical trial data was refreshed on June 15, 2020 (CT615).

For the E918 experimental outcomes (Fig. 4*A*), the best area under the curve (AUC) of 0.63 is provided by P1, followed by P2 (AUC = 0.58) and P3 (AUC = 0.58). For CT415 (Fig. 4*B*), we observed particularly strong predictive power for the four AI-based pipelines (AUC of 0.73-0.76), followed by proximity P1 (AUC = 0.57) and P2 (AUC = 0.56).

The goal of drug repurposing is to prioritize all available drugs, allowing us to limit experimental efforts only to the top-ranked compounds: hence, improve efficiency and resource utilization. Therefore, measuring the number of positive outcomes at the top of the list offers a better measure to evaluate the predictive power than the AUC. Thus, the most appropriate performance metric is the number of positive outcomes among the top *K*-ranked drugs (precision at *K*), and the fraction of all positive outcomes among the top *K*-ranked drugs (recall at *K*). For the E918 dataset (Fig. 4*C*), A2 ranks 9 S&W drugs among the top 100, followed by P1 (7 drugs), and A3 and A4 (6 drugs). We observe similar trends for recall (Fig. 4*E*): the A2 pipeline ranks 11.7% of all positive drugs in the top 100, while P1 selects 9%. Finally, A1 ranks 12 drugs currently in clinical trials among the top 100 in CT415, followed by A3 (11 drugs) and A2 (10 drugs), trends that are similar for recall (Fig. 4*F*).

Taken together, our first key results have the finding that while most algorithms show statistically significant predictive power (*SI Appendix*, Section 5.1 and Tables S1 and S2), they have different performance on the different ground truth datasets: the AI pipeline offers strong predictive power for the drugs selected for clinical trials, while proximity offers better predictive power for the E918 experimental outcomes. While together the 12 pipelines identify 22 positive drugs among the top 100, none of the pipelines offer consistent superior performance for all outcomes, prompting us to develop a multimodal approach that can extract the joint predictive power of all pipelines.

### Multimodal Approach for Drug Repurposing.

Predictive models for drug repurposing are driven by finite experimental resources that limit downstream experiments to those involving a finite number (*K*) of drugs. How do we identify these *K* drugs to maximize the positive outcomes of the tested list (19)? With no initial knowledge as to which of the *N*_*p*_ = 12 predictive pipelines offer the best predictive power, we could place equal trust in all by selecting the top *K/N*_*p*_ drugs from each pipeline (Union list). We compared this scenario with an alternative strategy that combines the predictions of the different pipelines. A widely used approach is to calculate the average rank of each drug over the *N*_*p*_ pipelines (29) (Average Rank list). The alternative is to search for consensus ranking that maximizes the number of pairwise agreements between all pipelines (16, 19). As the optimal outcome, called the Kemeny consensus (29), is *NP*-hard to compute, we implemented three heuristic rank aggregation algorithms that approximate the Kemeny consensus: Borda’s count (30), the Dowdall method (31), and CRank (16). For example, if the resources allow us to test *K* = 120 drugs, we ask which ranked list offers the best precision and recall at 120: the Union list collecting the top 10 predictions from the 12 pipelines; the top 120 predictions of Average Rank, Borda, Dowdall, or CRank; or the top 120 drugs ranked by an individual pipeline.

We found that Average Rank offers the worst performance, trailing the predictive power of most individual pipelines (Fig. 4 *G* and *H*). The Union List and Dowdall offer better outcomes, but trail behind the best performing individual pipelines (E918, CT415). Borda has a strong predictive performance for E918, but not for CT415. In contrast, CRank, which relies on Bayesian factors, offers a consistently high predictive performance for all datasets and most *K* values. CRank performs equally well for two other datasets: a manually curated prospective list E74 (described in *Discussion*) and the list of clinical trials updated on June 15, 2020 (C615) (*SI Appendix*, Fig. S8). In other words, we found that CRank extracts the cumulative predictive power of all methods, matching or exceeding the predictive power of the individual pipelines across all datasets, representing our second key result. Its persistent performance indicates that an unsupervised multimodal approach can significantly improve the hit rate over individual prediction algorithms. It also suggests that in the absence of a ground truth, the Kemeny consensus, which seeks a ranking with the smallest number of pairwise disagreements between the individual pipelines, represents an effective and theoretically principled strategy when each pipeline carries some predictive power.

### Confirmation in Human Cell Lines.

Of the 200 drugs ranked by CRank, 13 had positive outcomes in VeroE6 cells, representing promising drugs candidates that need to be tested further in human cells to confirm their clinical relevance. As chloroquine and hydroxychloroquine have been tested repeatedly in the literature, we experimentally tested the remaining 11 drugs in Huh7 cells, in a nine-point dilution series from 25 μM to 100 nM. Of the 11 compounds tested, auranofin, azelastine, digoxin, and vinblastine show very strong anti–SARS-CoV-2 response; fluvastatin displays a weaker response; and methodextrate is effective only at the highest concentration. Altogether, we found that 6 of the 11 drugs show potential for treating SARS-CoV2 infection (*SI Appendix*, Figs. S6 and S7).

Inspecting the CRank list and the experimental outcomes, we found three highly ranked drugs with strong outcomes, but not yet in clinical trials (Table 2): azelastine (CRank #10, S), an antihistamine used to treat allergic upper airway symptoms; and digoxin (CRank #33, S), used to treat heart failure and atrial fibrillation. Finally, in particular, auranofin (CRank #118, S), used to treat rheumatoid arthritis, also shown to reduce several microbial infections by altering cell redox state (32) and used to treat asthma, shows exceptionally strong response in human cells at clinically relevant concentrations. Our findings, coupled with extensive experience in their use in the clinical community, argue for their consideration in clinical trials. Other highly ranked candidates include methotrexate (CRank #32, S), which impairs folate metabolism and attenuates host inflammatory response in autoimmune diseases. This latter mechanism argues that methotrexate is likely to be effective at the other end of the disease spectrum (i.e., in the face of profound hyperimmune response to the infection).

**Table 2.** CRank predictions for drug repurposing

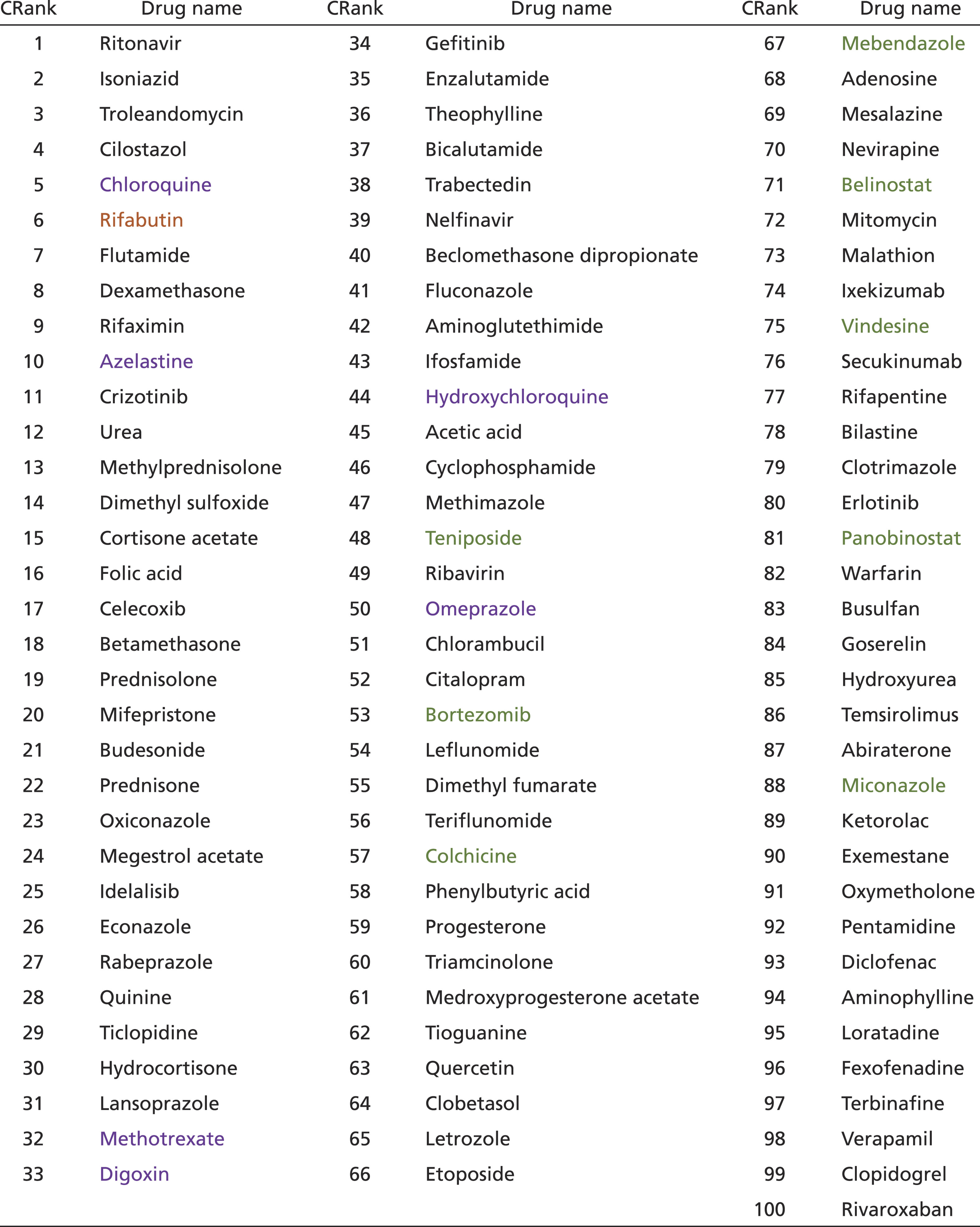

Top 100 consensus predictions of the drug-repurposing pipelines aggregated using the CRank algorithm. The top 100 drugs contain 9 drugs with positive experimental outcomes (S&W), 3 of which are among the top 10 drugs. Drugs in purple correspond to strong outcomes (S), in orange to weak outcomes (W), in green to cytotoxic drugs, and nonhighlighted drugs have shown no effect (N) in VeroE6 cells.

### Network Effects.

Most computationally informed drug-repurposing methods rely on docking patterns and, hence, are limited to compounds that bind either to viral proteins or to the host targets of the viral proteins (21) (Fig. 1*C*). A good example is remdesivir, a direct-acting antiviral that inhibits viral RNA polymerase (33, 34). In contrast, our pipelines can also identify drugs that target host proteins to induce network-based perturbations, some of which are likely to alter the virus’s ability to enter the cell or replicate within it. In the intact host, these drugs may also act via other mechanisms [such as the antiinflammatory effects of corticosteroids like dexamethasone (35)], which can only be assessed in animal models or in human trials.

We find that only one of the 77 S&W drugs are known to target directly a viral protein binding target: amitriptyline, which targets SIGMAR1, the target of the NSP6 SARS-CoV-2 protein. In other words, 76 of the 77 drugs that showed efficacy in our experimental screen are “network drugs,” achieving their effect by perturbing the host subcellular network, representing our third key finding. Indeed, as network drugs do not target viral proteins or their host targets, they cannot be identified using traditional binding-based methods; yet, they are successfully prioritized by network-based methods.

Searching for common mechanistic or structural patterns that could account for the efficacy of the 77 S&W drugs, we explored their target and pathway enrichment profiles (*SI Appendix*, Figs. S6 and S7), as well as their reported mechanisms of action, failing to identify statistically significant features shared by most S&W drugs. This failure is partly explained by the diversity of the S&W drugs (Dataset S8), containing antipsychotics (nine S and four W), serotonin receptor agonists (three W), nonsteroidal antiinflammatory drugs (two W), angiotensin receptor blockers (two W), tyrosine kinase inhibitors (five S), statins (one W and two S), and others. We did, however, find a connected component formed by the targets of the drugs that were effective viral inhibitors (Fig. 3*B*), suggesting that even though we failed to find a strong mechanistic pattern common to most drugs, we did find a neighborhood in the interactome that may be implicated with the inhibition of viral replication. Note also that each pipeline relies on different network features, and therefore, captures different reasons as to why a drug may alter the outcome of a disease. Indeed, the proximity pipeline identifies the physical interactions that connect the drug targets to the disease module (*SI Appendix*, Section 3), offering specific, experimentally testable predictions of the drug’s mechanism in the context of the disease (36, 37). While the AI and the Diffusion pipelines are not explicit about why they make their predictions, we were able to extract the predictive subgraphs that collect the interactions that may contribute to the therapeutic a mechanism (*SI Appendix*, Section 3). As CRank extracts its predictive power from the network, we hypothesized that network-based patterns may help distinguish the S&W drugs from the N drugs. Indeed, we found that the targets of the 37 S drugs form a statistically significant large connected component (*z*-score = 2.05), indicating that these targets agglomerate in the same network neighborhood. We observe the same pattern for the targets of the 40 W drugs (*z*-score = 3.42). The negative network separation between the S and W drug targets (S_SW_ = −0.69) indicates that, in fact, the S and the W drugs target the same network neighborhood. To characterize this neighborhood, we measured the network-based proximity of the targets of the S, W, and N drug classes to the SARS-CoV-2 targets. We found that compared to random expectation, the N drug targets are far from the COVID-19 module (Fig. 3*C*), while the S and W drug targets are closer to the COVID-19 disease module than expected by chance. The magnitude of the effect is also revealing: The S drug targets are closer than the W drug targets, suggesting that network proximity is a positive predictor of a drug’s efficacy.

Taken together, our analyses suggest that S&W drugs are diverse, and lack pathway-based or mechanistic signatures that distinguish them. We did find, however, that S&W drugs target the same interactome neighborhood, located in the network vicinity of the COVID-19 disease module, potentially explaining their ability to influence viral effects on host cells, and the effectiveness of network-based methodologies to identify them.

## Discussion

A recent in vitro screen (1) of 12,000 compounds in VeroE6 cells identified 100 compounds that inhibit viral infectivity. Yet, only 39% of the 12,000 compounds tested are Food and Drug Administration (FDA) approved, the rest being in the preclinical or experimental phase, years from reaching patients. In contrast, 96% of the 918 drugs prioritized and screened here are FDA approved and, hence, could be moved rapidly to clinical trials. Brute force screening does, however, offer an important benchmark: Its low hit rate of 0.8% highlights the need to prioritize resources toward the most promising compounds. Indeed, the unsupervised CRank offers an order-of-magnitude higher (9%) hit rate among the top 100 drugs, and the top 800 of the 6,340 drugs prioritized by CRank contains 58 of the 77 S&W drugs (Fig. 4 *G* and *H*). The hit rate can be further increased by expert knowledge and curation. To demonstrate this point, we mimicked the traditional drug-repurposing process whereby a physician–scientist manually inspected the top 10% of the CRank consensus ranking on April 15, removing drugs with known significant toxicities in vivo and lower-ranked members of the same drug class, and arrived at 74 drugs available for testing. Using the experimental design described above but over a wider range of doses (0.625 – 20 µM, 0.2 multiplicity of infection), we screened these 74 compounds separately from the E918 list, and found 39 N, 10 W, and 11 S outcomes (Dataset S9). The resulting 28% enrichment of S&W drugs suggests that in the case of limited resources, outcomes are maximized by combining algorithmic consensus ranking with expert knowledge. Finally, the real value of the predictive approach is demonstrated after selecting drugs that in the nonhuman primate screen had a positive outcome for a second human screen, resulting in a success rate of 62%, helping us identify six drugs could be easily repurposed for treating the SARS-CoV2 infection.

Taken together, the methodological advances presented here not only suggest potential drug candidates for COVID-19, but offer a principled algorithmic toolset to identify future treatments for diseases underserved by the cost and the timelines of conventional de novo drug discovery processes. As only 918 of the 6,340 drugs prioritized by CRank were screened, a selection driven by compound availability, many potentially efficacious FDA-approved drugs remain to be tested. Finally, it is also possible that some drugs that lacked activity in VeroE6 cells may nevertheless show efficacy in human cells, like loratadine (rank #95, N), which inhibited viral activity in the human cell line Caco-2 (38). Ritonavir, our top-ranked drug, also showed no effect in our screen, despite the fact that over 42 clinical trials are exploring its potential efficacy in patients. In other words, some of the drugs highly ranked by CRank may show efficacy, even if they are not among the 77 S&W drugs with positive outcomes. Note that a drug can have inhibitory effect in vitro that might not replicate in vivo, as observed for chloroquine and hydroxychloroquine (39). Moreover, drug combinations could increase the potency of some drugs (11), and given a synergistic effect, could also improve outcomes.

COVID disease is the product of damage by the virus itself and damage by immune overreaction (cytokine storm). As the assay used for the experimental screening only detects the inhibition of the viral replication cycle, an immunomodulatory drug that reduces the cytokine storm without interfering with virus replication would not show up as a hit in our screen. However, we identify drugs that reduce the viral load enough such that the immune system is not overstimulated, potentially lowering the chance of a cytokine storm. Our predictions could be further improved by leveraging drug–target binding predictions, along with the experimentally known bindings already included, by the use of gene-expression datasets for different cell lines perturbed by the explored drugs. Finally, Clinical Trials data outcomes could also be used for drug selection or ranking. Note that such additional data need to be available for all drugs, not just for selected examples.

## Materials and Methods

### Human Interactome, SARS-CoV-2, and Drug Targets.

The human interactome was assembled from 21 public databases that compile experimentally derived protein–protein interactions (PPI) data: 1) binary PPIs, derived from high-throughput yeast two-hybrid experiments, three-dimensional protein structures; 2) PPIs identified by affinity purification followed by mass spectrometry; 3) kinase substrate interactions; 4) signaling interactions; and 5) regulatory interactions. The final interactome used in our study contains 18,505 proteins, and 327,924 interactions between them. We retrieved interactions between SARS-CoV-2 human proteins detected by Gordon et al. (21), and drug–target information from the DrugBank database. A detailed description on the datasets can be found in *SI Appendix*, Section 1.1.

### Graph Convolutional Networks.

We designed a graph neural network for COVID-19 treatment recommendations (14), where nodes represent three distinct types of biomedical entities (i.e., drugs, proteins, diseases), and labeled edges represents four types of edges between the entities (PPIs, drug-target associations, disease–protein associations, and drug disease treatments). A detailed description of the method is presented in *SI Appendix*, Section 2.1.

### Diffusion State Distance.

The diffusion state distance (17) algorithm uses a graph diffusion to derive a similarity metric for pairs of nodes that takes into account how similarly they impact the rest of the network. A detailed description of the method and its implementation is in *SI Appendix*, Section 2.2.

### Network Proximity.

We calculated the proximity of the SARS-CoV2 targets to drug targets using the proximity (11). A detailed description of the method and randomization can be found in *SI Appendix*, Section 2.3.

## Supplementary Material

Supplementary File

Supplementary File

Supplementary File

Supplementary File

Supplementary File

Supplementary File

Supplementary File

Supplementary File

Supplementary File

Supplementary File

Supplementary File

Supplementary File

Supplementary File

## Data Availability

All study data are included in the article and supporting information. The code is available in Github at https://github.com/Barabasi-Lab/COVID-19.
