# Supplementary material for: Network medicine framework for identifying drug-repurposing opportunities for COVID-19
Source: Proc Natl Acad Sci U S A. 2021 Apr 27;118(19):e2025581118. doi: 10.1073/pnas.2025581118 (PMC8126852; doi:10.1073/pnas.2025581118)
Supplement: Supplementary File [file pnas.2025581118.sapp.pdf]

Supplementary Information for

Network Medicine Framework for Identifying Drug Repurposing Opportunities for COVID-19.

Deisy Morselli Gysi, Ítalo Do Valle, Marinka Zitnik, Asher Ameli, Xiao Gan, Onur Varol, Susan Dina Ghiassian, JJ Patten, Robert Davey, Joseph Loscalzo, and Albert-Lászlo Barabási*

* Albert-László Barabási. **Email:** a.barabasi@northeastern.edu.

This PDF file includes:

Supplementary text

Figures S1 to S10

Tables S1 to S2

Legends for Datasets S1 to S12

SI References

Other supplementary materials for this manuscript include the following:

Datasets S1 to S12

1 Network-Based Drug Repurposing For COVID-19

1.1 Human Interactome and SARS-CoV-2 and Drug Targets.

The human interactome was assembled from 21 public databases that compile experimentally derived protein-protein interaction (PPI) data: 1) binary PPIs, derived from high-throughput yeast-two hybrid (Y2H) experiments (HI-Union (1)), three-dimensional (3D) protein structures (Interactome3D (2), Instruct (3), Insider (4)), or literature curation (PINA (5), MINT (6), LitBM17 (1), Interactome3D, Instruct, Insider, BioGrid (7), HINT (8), HIPPIE (9), APID (10), InWeb (11)); 2) PPIs identified by affinity purification followed by mass spectrometry present in BioPlex (12), QUBIC (13), CoFrac (14), HINT, HIPPIE, APID, LitBM17, and InWeb; 3) kinase substrate interactions from KinomeNetworkX (15) and PhosphoSitePlus (16); 4) signaling interactions from SignaLink (17) and InnateDB (18); and 5) regulatory interactions derived by the ENCODE consortium. We used the curated list of PSI-MI IDs provided by Alonso-Lopez et. al. (2019) (10) for differentiating binary interactions among the several experimental methods present in the literature-curated databases. For InWeb, interactions with curation scores < 0.175 (75th percentile) were not considered. All proteins were mapped to their corresponding Entrez ID (NCBI), and the proteins that could not be mapped were removed. The final interactome used in our study contains 18,505 proteins and 327,924 interactions (Dataset 2). We retrieved interactions between 26 SARS-CoV-2 proteins and 332 human proteins reported by Gordon, et. al. (2020) (Dataset 1). We retrieved drug target information from the DrugBank database, which contains 24,609 interactions between 6,228 drugs and their 3,903 targets, and drug target interaction data curated from the literature for 25 drugs (Dataset 3). We also obtained from the DrugBank database differentially expressed genes (DEGs) identified by exposure of drugs to different cell lines (Dataset 4). The Largest Connected Component (LCC) of human proteins that bind to SARS-CoV-2 proteins was calculated using a degree-preserving approach, which prevents the repeated selection of the same high degree nodes, setting 100 degree bins in 1,000 realizations.

1.2 Lung Gene Expression (Fig 2A).

We evaluated gene expression in the lung by using the GTEX database, considering genes with a median count lower than 5 transcripts (raw counts) as not expressed.

1.3 Disease Comorbidities.

Pre-existing conditions worsen prognosis and recovery of COVID-19 patients. Previous work showed that the disease relevance of human proteins targeted by a virus can predict the signs, symptoms, and diseases caused by that pathogen. This prompted us to identify diseases whose molecular mechanisms overlap with cellular processes targeted by SARS-CoV-2, allowing us to predict potential comorbidity patterns (25–27). We retrieved 3,173 disease-associated genes for 299 diseases (28), finding that 110 of the 332 proteins

targeted by SARS-CoV-2 are implicated in other human diseases; however, the overlap between SARS-CoV-2 targets and the pool of the disease-associated genes was not statistically significant (Fisher's exact test; FDR-BH p_{adj} -value> 0.05). We evaluated the network-based overlap between the proteins associated with each of the 299 diseases and the host protein targets of SARS-CoV-2 using the S_{vb} metric (28), where $S_{vb} < 0$ signals a network-based overlap between the SARS-CoV-2 viral targets v and the gene pool associated with disease b. We found that $S_{vb} > 0$ for each disease, indicating that COVID-19 disease module does not directly overlap with any major disease module (Fig. S1 and Dataset 5). The diseases closest to the COVID-19 disease module (smallest S_{vb}) included several cardiovascular diseases and cancers, whose comorbidity in COVID-19 patients is well documented (29–31) (Fig. S2). The same metric predicted comorbidity with neurological diseases, in line with our observation that the host protein targets are expressed in the brain (Dataset 5).

In summary, we found that the SARS-CoV-2 host protein targets do not overlap with proteins associated with any major diseases, indicating that a potential COVID-19 treatment cannot be derived from the arsenal of therapies approved for a specific disease. These findings argue for a strategy that maps drug targets without regard to their localization within a particular disease module. However, the disease modules closest to the SARS-CoV-2 viral targets are those with noted comorbidity for COVID-19 infection, such as pulmonary and cardiovascular diseases, and obesity. We also found multiple network-based evidences linking the virus to the nervous system, a less explored comorbidity, consistent with the observations that many infected patients initially lose olfactory function and taste (32), and 36% of patients with severe infection who require hospitalization have neurological manifestations.

2 Drug Repurposing Prediction Algorithms

To obtain drug repurposing predictions we implemented three algorithmic approaches: i) Artificial Intelligence Based Algorithm (A1-A4); ii) Diffusion-Based Algorithms (D1-D5) and iii) Proximity Based Algorithms (P1-P3). The AI algorithm is a graph neural network (GNN) architecture that takes as input a multimodal graph with three types of nodes (representing drugs, proteins, and diseases) and edges capturing different types of interactions between these nodes. The algorithm generates embedding vectors of drug and disease nodes, which are then used to predict drug scores, representing how promising a given drug is for COVID-19. The diffusion-based algorithms are inspired by the diffusion state distance (DSD) (42). They use a diffusion property to define a similarity metric for node pairs, taking into account how similar the nodes are in terms of how they affect the rest of the network. Once pairwise similarity scores between all nodes are obtained, we calculate how similar drug targets are to the pool of SARS-CoV-2 proteins. This indicates how likely drug targets reverse the impact of the SARS-CoV-2 proteins. Finally, the proximity measure (21) is based on the average shortest path from a drug target to a SARS-CoV-2 target.

2.1 Artificial Intelligence Based Algorithm (A1-A4).

We designed a graph neural network for COVID-19 treatment recommendations based on a previously developed graph neural network (GNN) architecture (33) (Fig. S3). The multimodal graph is a heterogeneous graph $G = (V, R)$ with N nodes $v_i \in V$ representing three distinct types of biomedical entities (i.e., drugs, proteins, diseases), and triplets, i.e., labeled edges $(v_i, r, v_j) \in R$ representing four semantically distinct types of edges r between the entities (i.e., protein-protein interactions, drug-target associations, disease-protein associations, and drug-disease indications).

COVID-19 drug treatment recommendation task: We cast the COVID-19 treatment recommendation problem as a link prediction task on the multimodal graph. The task was to predict new edges between drug and disease nodes such that a predicted link between a drug node v_i and a disease node v_j should carry the meaning that the drug v_i is indicated for the disease v_j (i.e., the drug has a known positive therapeutic effect in patients with the disease, e.g., COVID-19). Parameters of the GNN model were optimized during training to maximize the model's ability to predict examples of known and approved drug-disease indications. This process produced embeddings for drug and disease nodes in the graph that were predictive of therapeutic indications, and we used the embeddings to construct ranked lists of candidate drugs for COVID-19.

Overview of graph neural architecture: Our graph neural network is an end-to-end trainable model for link prediction on the multimodal graph and has two main components: (1) an encoder: a graph convolutional network operating on G and producing embeddings for nodes in G; and (2) a decoder: a model optimizing embeddings such that they are predictive of known drug-disease indications. The neural message-passing encoder took as input a graph G and produced a node d-dimensional embedding $z_i \in R^d$ for every drug and disease node in the graph. We used the encoder (33) that learned a message-passing algorithm (34) and aggregation procedure to compute a function of the entire graph that transformed and propagated information across graph G (34). The graph convolutional operator took into account the first-order neighborhood of a node and applied the same transformation across all locations in the graph. Successive application of these operations then effectively convolved information across the Kth order neighborhood (i.e., embedding of a node depends on all the nodes that are at most K steps away), where K is the number of successive operations of convolutional layers in the neural network model. The graph convolutional operator takes the form

$$h_i^{(k+1)} = \phi\left(\sum_r \sum_{j \in N_r^i} \alpha_r^{ij}\, W_r^{(k)} h_j^{(k)} + \alpha_r^i h_i^{(k)}\right), \qquad (1)$$

where $h_i^{(k)} \in R^{d\,(k)}$ is the hidden state of node v_i in the kth layer of the neural network with $d\,(k)$ being the dimensionality of this layer's representation, r is an edge type, matrix $W_r^{(k)}$ is an edge-type specific

parameter matrix, ϕ denotes a non-linear element-wise activation function (*i.e.*, a rectified linear unit), and α_r denote attention coefficients (35). To arrive at the final embedding $z_i \in R^d$ of node v_i, we compute its representation as $z_i = h_i^{(k)}$. Next, the decoder takes node embeddings and combines them to reconstruct labeled edges in G. In particular, the decoder scores a (v_i, r, v_j) triplet through a function g whose goal is to assign a score $g\ (v_i, r, v_j)$ representing how likely it is that drugs v_i will treat disease v_j (i.e., r denotes an 'indication' relationship) (35).

Training a graph neural network model: During model training, we optimized model parameters using the max-margin loss functions to encourage the model to assign higher probabilities to successful drug indications (v_i, r, v_j) than to random drug-disease pairs. We took an end-to-end optimization approach that jointly optimized over all trainable parameters and propagated loss function gradients through both the encoder and the decoder. To optimize the model, we trained it for a maximum of 100 epochs (training iterations) using the Adam optimizer (36) with a learning rate of 0.001. We initialized weights using the initialization described in (37). To make the model comparable to other drug repurposing methodologies in this study, we did not integrate additional side information into node feature vectors; instead, we used one-shot indicator vectors (38) as node features. For the model to generalize well to unobserved edges, we applied a regular dropout (39) to hidden layer units (Eq. (10)). In practice, we used efficient sparse matrix multiplications, with complexity linear in the number of edges in G, to implement the model. We used a 2-layer neural architecture with $d_1 = 32$, $d_2 = 32$, $d_i = 128$ hidden units in input, output, and intermediate layer, respectively; a dropout rate of 0.1; and a max-margin of 0.1. We used mini-batching (40) by sampling triplets R from the multimodal graph G. That is, we processed multiple training mini-batches (mini-batches are of size 512), each obtained by sampling only a fixed number of triplets, resulting in dynamic batches that changed during model training.

Constructing ranked lists of candidate drugs for COVID-19: We generated four lists of candidate drugs for COVID-19. To generate the lists, we used embeddings returned by the graph neural network, in particular, embeddings learned for nodes representing either COVID-19 or drugs in multimodal graph G. The embedding vectors for diseases and drugs are provided in Dataset 6 and Dataset 7, respectively. The pipeline A1 searches for drugs that are in the vicinity of the COVID-19 disease by calculating the cosine distance between COVID-19 and all drugs in the decoded embedding space (41). The decoding is based on the $N = 10$ nearest neighboring nodes in the embedding space, with a minimum distance between nodes of $D = 0.25$. The pipeline A2 prevents that nodes in the decoding embedding space from packing together too closely, by using $D = 0.8$ and keeping N unchanged. These constraints push the structures apart into softer, more general features, offering a better overarching view of the embedding space at the loss of the more detailed structure. Pipeline A3 forces the decoding to concentrate on the very local structure by using N = 5, to explore a smaller neighborhood, while setting the minimum distance at a midrange point of D = 0.5. Pipeline A4 focuses on a broader view of the embedding space by setting N=10

and D = 1. Finally, to obtain lists of candidate drugs, each pipeline ranked drugs based on the pipeline-defined distances of drugs to COVID-19 (Fig. S3). Intuitively, parameter N constrained the size of the local neighborhood each pipeline looked at in the embedding space when calculating the distances, and parameter D controlled how tightly the pipeline was allowed to pack the embeddings together.

2.2 Diffusion-Based Algorithms (D1-D5).

The diffusion state distance (DSD) (42) algorithm uses a graph diffusion property to derive a similarity metric for pairs of nodes that takes into account how similarly they affect the rest of the network. We calculate the expected number of times $He\ (A, B)$ that a random walker starting at node A visits node B, representing each node by the vector (42):

$$He\ (V_i) = [He(V_i, V_1), He(V_i, V_2), He(V_i, V_3), \dots, He(V_i, V_n)], \qquad (2)$$

which describes how a perturbation initiated from that node affects other nodes in the interactome. The similarity between nodes A and B is provided by the L1-norm of their corresponding vector representations:

$$DSD(A, B) = ||He(A) - He\ (B)||. \qquad (3)$$

Inspired by the DSD, we developed five new metrics to calculate the impact of drug targets T on the SARS-CoV-2 targets V. The first (Pipeline D1) is defined as:

$$I_{DSD}^{min} = \frac{1}{||T||} \sum_{t \in T} \min_{v \in V} DSD\ (t, v), \qquad (4)$$

where $DSD\ (s, t)$ represents the diffusion state distance between nodes t and v. Since the L1-norm of two large vectors may result in loss of information (43), we also used the metrics (Pipeline D2):

$$I_{KL}^{min} = \frac{1}{||T||} \sum_{t \in T} \min_{v \in V} KL\ (t, v) \qquad (5)$$

and (Pipeline D3):

$$I_{KL}^{med} = \frac{1}{||T||} \sum_{t \in T} \operatorname*{median}_{v \in V} KL\ (t, v), \qquad (6)$$

where KL is the Kullback-Leibler (KL) divergence between the vector representations of the nodes t and s. Finally, to provide symmetric measures, we tested the metrics (Pipeline D4):

$$I_{JS}^{min} = \frac{1}{||T||} \sum_{t \in T} \min_{v \in V} JS\ (t, v) \qquad (7)$$

and (Pipeline D5)

$$I_{JS}^{med} = \frac{1}{||T||} \sum_{t \in T} \underset{v \in V}{\text{median}} JS\ (t, v). \qquad (8)$$

where JS is the Jensen-Shannon (JS) divergence between the vector representations of nodes t and s. All five measures assume $t \neq s$.

2.3 Proximity Algorithm (P1-P3).

Given V, the set of COVID-19 virus targets, T, the set of drug targets, and $d\ (v, t)$, the shortest path length between nodes $v \in V$ and $t \in T$ in the network, we define (21):

$$d_c(V, T) = \frac{1}{||T||} \sum_{t \in T} \min_{v \in V} d\ (v, t). \qquad (9)$$

We determined the expected distances between two randomly selected groups of proteins, matching the size and degrees of the original V and T sets. To avoid repeatedly selecting the same high degree nodes, we use degree-binning (21). The mean $\mu_{d\ (V,T)}$ and standard deviation $\sigma_{d\ (V,T)}$ of the reference distribution allows us to convert the absolute distance $d_c$to a relative distance Z_{dc}, defined as:

$$Z_{d_c} = \frac{d_c - \mu_{d_c}\ (V,T)}{\sigma_{d_c}\ (V,T)}. \qquad (10)$$

We implemented three versions of the proximity algorithm: 1) relying on all drug targets (P1); 2) ignoring drug targets identified as drug carriers, transporters, and drug-metabolizing enzymes – and therefore removing all proteins that had functions involved in drug delivery and metabolism (P2); and 3) based on differentially expressed genes (DEGs) identified by exposure of each drug to cultured cells, which was obtained from DrugBank's compilation of 17,222 DEGs linked to 793 drugs in multiple cell lines (Dataset 4). P2 aims to understand if the role of proteins involved in drug delivery and drug metabolism can improve the prediction power of the proximity measure and P3 aims to understand if the use of differentially expressed genes under the presence of the drug – instead of binding information – was able to improve the proximity's accuracy.

3 Network Properties of Prediction Algorithms

3.1 Explanatory Subgraphs.

For each pipeline, we identified "explanatory subgraphs" to help understand the predictions made by the respective pipeline. The key idea was to summarize where in the data the pipeline seeks evidence for their predictions. Given a particular prediction, an explanatory subgraph is a small sub-network of the entire

network considered by the pipeline that is most influential for the prediction and contributes most to the predictive power. For the proximity method (P), the explanatory subgraphs can be derived exactly, representing the set of nodes contributing to proximity. For the artificial intelligence-based methods (A), the subgraphs were extracted using a GNN Explainer algorithm (44). GNNExplainer specifies an explanation as a subgraph of the entire network the GNN was trained on, such that the subgraph maximizes the mutual information with the GNN's prediction. This is achieved by formulating a mean field variational approximation and learning a real-valued graph mask, which selects the important subgraph using counterfactual reasoning. For the diffusion method, we first identified the SARS-CoV-2 targets (seeds) that have the maximum (or median, depending on the pipeline) similarity with the drug targets under consideration. Once the seeds are identified for each drug target, we extract the vector representation of the target and the corresponding seeds. Each element of these vectors corresponds to a node in the network:

$$t: [r_1, r_2, r_3, \ldots, r_n]$$

$$s: [w_1, w_2, w_3, \ldots, w_n]$$

Each pipeline performs an element-wise comparison of these two vectors to calculate similarity values, defined as similarity terms, using:

$$term_i^{DSD}(t, s) = |r_i - w_i| \quad (11)$$

$$term_i^{KL}(t, s) = r_i \log\left(\frac{r_i}{w_i}\right) \quad (12)$$

$$term_i^{JS}\ (t, s) = \frac{1}{2}\left[r_i \log\left(\frac{r_i}{m_i}\right) + w_i \log\ (\frac{w_i}{m_i})\right], m_i = \frac{r_i + w_i}{2} \quad (13)$$

These distance similarity terms collectively contribute to each drug's ranking score. Among all 18,446 nodes, we are only interested in those whose variations lead to the current ranking (drug prediction scores). Therefore, we applied a feature selection algorithm to eliminate the network nodes (features) that do not contribute to the predicted scores (outcomes). This task is done by training a regression tree model (DecisionTreeRegressor model, from Python 3 scikit-learn package) where feature values are the similarity terms (as defined above) between drug targets and the corresponding seeds. This resulted in 2,507 important features for pipeline D1 (DSD-min), 2198 for D2 (KL-min), 2,263 for D3 (KL-med), 1,655 for D4 (JS-min), and 1,817 for D5 (JS-med). Important features are those with non-zero importance value as characterized by the Regressor model.

Once the important features/nodes are extracted, we search this space to identify the explanatory network of each set of drug targets. To do so, we rank the similarity terms of each target and the corresponding

seeds on the space of important features and identify the nodes with the highest contribution to the similarity measure such that they satisfy the following equation:

$$\log_{10}\left(\frac{l}{term_i}\right) \leq 1, l = \max(term_i), i \in V \quad (14)$$

If a drug has multiple targets or if each target has multiple corresponding seeds (seeds with the same similarity to a target), the results are aggregated. The explanatory network of a target that happens to be a seed is that seed itself.

Fig. S4 shows the similarities and differences among the explanatory subgraphs of the different prediction pipelines.

3.2 Complementarity of Prediction Algorithms (Fig 2C).

To investigate the complementarity among the prediction algorithms, for each drug we measured the network separation S_{G-d} between the explanatory subgraph G and the drug's targets (d), and the separation S_{G-v} between G and the 332 SARS-Cov2 viral targets (v) capturing the disease module. Each drug has twelve subgraphs, each corresponding to one of the twelve pipelines. A total of 320 drugs, for which all pipelines have predictive subgraph and separation values, are shown in Fig. S5. Proximity Pipeline 3 uses differentially expressed genes as input drug data; thus, for proximity P3 we computed the separation between the subgraph and the differentially expressed genes. The figure shows complementarity patterns between methods: the AI pipelines extracts their predictions from subgraphs that overlap with the drug targets ($S_{G-d} < 0$), but are separated from the COVID-19 module ($S_{G-v} > 0$); proximity-based methods show the opposite pattern – for most of the predictive subgraphs the overlap with the COVID-19 module is apparent ($S_{G-v} < 0$); by contrast, diffusion-based predictive subgraphs avoid both the drug targets and the disease module ($S_{G-d} > 0, S_{G-v} > 0$).

4 Experimental Validation

4.1 Cell Cultivation and Viruses Used.

VeroE6 cells were obtained from ATCC (Manassas, VA, USA) and maintained in DMEM supplemented with 10% fetal bovine serum (FBS) at 37°C in a humidified CO2 incubator. The virus strain used was isolated from a traveler returning to Washington State, USA, from Wuhan, China, (USA-WA1/2020) and was obtained from BEI resources (Manassas, VA, USA). The virus stock was passaged twice on VeroE6 cells by challenging the cells at an MOI of less than 0.01 and incubating until cytopathology was seen (typically 3 days after inoculation). A sample of the culture supernatant was sequenced by next generation

sequencing (NGS) and was consistent with the original isolate without evidence of other viral or bacterial contaminants. The virus stock was stored at -80°C. The virus stock was serially passaged as above several times further on Huh7 cells for use in Huh7 cell infection assays.

High Throughput Virus Infection Inhibition Assay (E918). To evaluate the efficacy of a large library of compounds against SARS-CoV-2 infection, a high throughput screen of >6700 compounds was performed as described in [Patten et al., 2020]. In short, compounds were pre-spotted into 384 well plates and diluted in culture medium before being added to VeroE6 cells. The dilution scheme was a four-point ten-fold series, with final concentrations ranging from 8uM to 8nM. Compounds were incubated on cells for more than an hour, then challenged with virus at an MOI of about 0.2. After a 1 – 1.5 day incubation, cells were treated with 10% buffered formalin for at least 6 hours, washed in PBS, and virus antigen stained with SARS-CoV-2 specific antibody (Sino Biologicals, MM05) together with Hoechst 33342 dye to stain cell nuclei. Plates were imaged by a Biotek Cytation 1 microscope, and automated image analysis was used to count total number of infected cells and total cell nuclei. CellProfiler software (Broad Institute, MA, USA) was used for image analysis using a customized processing pipeline (available upon request to RAD). Infection efficiency was calculated as the ratio of infected cells to total cell nuclei, and was normalized to negative controls. Loss of cell nuclei was used to flag treatments suggestive of host cell toxicity. Compounds were classified by DRC as described below. The assay was performed in duplicate.

4.2 Follow Up Virus Infection Assay (E74).

For further evaluation of small molecule efficacy against infection with wild type SARS-CoV-2 virus, compounds were first dissolved to 10 mM in DMSO and then diluted into culture medium before addition to cells. The compound stock was added to VeroE6 cells incubated for a minimum of 1 hour and then challenged with virus at a MOI of about 0.2. Dosing ranged from a final concentration of 25 μM down to 0.2 μM in a two-fold dilution series. As a positive control, 5 μM E-64 was used as it was previously reported to inhibit SARS-CoV-2 infection (Hoffman et al. 2020). Negative controls were <0.5% DMSO. Plates were processed as described above. Each assay was performed in duplicate in 384 well plates.

4.3 Drug-Response Classification.

The classification of the drug-response outcomes was done using a drug response curve (DRC) model (45). We used the R package drc (46) to calculate the DRCs using a log-logistic model with four parameters (hill, IC50, min, and max). Each drug-response was classified in two steps: first, inspecting toxicity, and later, evaluating the drug effect on the inhibition of viral proliferation.

To inspect the cytotoxicity, we first estimated the model parameters using as response variable the nuclei count in the treated cells, normalized by the nuclei count in the controls. We tested the dose-response

effect for all drugs using a χ^2 test for goodness of fit and drugs with $p < 0.01$ (Bonferroni correction) were defined as cytotoxic, with the exception of drugs demonstrating toxicity only at the highest dose. To evaluate inhibition of viral replication, we used as response for the DRC model the number of infected cells in the treated samples normalized by the controls. For that, a drug was considered to have a dose-response effect by using a χ^2 test for goodness of fit ($p < 0.01$, Bonferroni correction), and the significant drugs were defined as Strong (S) or Weak (W) if the viral reduction was greater than 80% and 50%, respectively. The drugs that did not meet the criteria for S or W were classified as no-effect (N). Finally, we classified drugs as cytotoxic (C) if their toxicity curves were greater than their viral proliferation curves in at least half of the doses tested.

4.4 Huh7 Confirmation.

We validated the outcomes for the top 200 ranked drugs with S&W response in the Huh7 cell line (human liver cell line). Drug dosing and infection were performed as described above, with remdesivir being used as a positive control. We found that six drugs had a positive response, and four of them (digoxin, fluvastatin, azelastine, and auranofin) are in a suitable dose bioavailability range (Fig. S6 and Fig. S7). Even though auranofin and azelastine showed tracing cytotoxicity in Huh7 cells, in high concentration, the dose range where they are reducing nuclei count are inside pharmacological usage range, moreover, auranofin has been in used for treating asthma. Furthermore, our prediction has been confirmed not only by our *in vitro* assays, but also by a contemporaneous set of *in vivo* experiments performed after we locked in our ranking results.

4.5 Biological Interpretation of Effective Drugs in E918 Dataset.

We observed 77 drugs that showed strong (S) or weak effects (W) in the high-throughput screening. There was no drug category (ATC Classification) that was enriched among the S, W, or S&W drugs (hypergeometric test FDR-BH padj > 0.05). To search for common patterns that could explain their bioactivity, we performed hierarchical clustering on the drug target profiles, failing to find binding patterns shared by all drugs (Fig. S8). Only four small groups of drugs are observed, documenting various degrees of shared targets (Fig. S8), three of which contain drugs from multiple categories, and one group consists of 7 nervous system-related drugs with similar target profiles. We also performed pathway enrichment analysis to identify biological processes shared across the targets of drugs with strong or weak effects. Among the 77 S&W drugs, 42 are located in three groups associated with common pathways, and 20 of these drugs are of diverse indications linked to transport and metabolism of different substrates. Eighteen are associated with pathways related to membrane receptors, most of them indicated for nervous system disorders, targeting G protein-coupled receptors such as ADRA1A, HTR2A, and HRH1 (Fig. S9). Taken together, neither the pathway nor the target analysis reveals patterns that could explain the efficacy of the 77 S&W drugs.

5 Statistical Validation

5.1 Performance Evaluation using ROC Curves, Precision, and Recall.

We examined whether positive drugs (e.g., strong-effect drugs) were ranked high by measuring the predictive power of each pipeline in terms of area under the ROC (Receiver Operating Characteristics) curve, precision, and recall. First, we calculated ROC (Receiver Operating Characteristics) curves and AUC (area under the curve) scores for model selection and performance analysis. The AUC score measures the separation between positive examples (e.g., drugs with strong or weak responses) and negative examples (e.g., drugs showing no-effect in experimental screening). For the ranked lists of drugs, we applied different thresholds to compute false-positive and true-positive rates to plot the ROC curves. Scores of AUC range between 0 and 1, where 1 corresponds to perfect performance and 0.5 indicates the performance of a random classifier. We used the R package ROCR for computing the AUC scores and ggplot2 plotting the ROC curves.

The AUC metric operates on the whole ranked list of drugs, and, thus, it does not directly reflect the ability of the method to prioritize the most promising drug candidates at the top of the list. To address this issue and account for unbalanced ground-truth information where negative examples vastly outnumber positives, we also considered hit-rate-based metrics to evaluate the quality of top-K drugs in each ranked list. Here, we evaluated performance at a given cut-off rank K, considering only the topmost predictions by the pipeline. In particular, we calculated the fraction of top-K ranked drugs that were positive outcomes (precision at K) and the fraction of all positive outcomes that were among the top-K ranked drugs (recall at K).

We considered four types of ground-truth information to evaluate prediction performance:

1) The outcome of the experimental screening of 918 compounds (E918 dataset, Dataset 8). We identified 806 no effect drugs, 40 with weak effect, and 37 with strong effect.
2) The outcome of the experimental screening of additional 74 compounds tested with a wider range of doses (0.625 – 20μM, 0.2 MOI) (E74 dataset, Dataset 9) (Fig. S10). The E74 dataset represents a subset of 81 compounds by a medical doctor among the top 10% of all drug predictions that were available for purchase. We identified 39 no effect drugs, 10 with weak effect, and 11 with strong effect.
3) 67 drugs that, as of April 2020, were in ongoing trials for COVID-19, obtained from the ClinicalTrials.gov website[**] (CT415 dataset, Dataset 10). ClinicalTrials.gov organizes COVID-19

[**] Clinical Trial Covid-19 selection: https://clinicaltrials.gov/ct2/results?cond=COVID-19

specific collection of all trials. Trial records consist of information on inclusion and exclusion criteria, details on drugs being tested, the scientific team behind the study, and funding agencies. We extract drug names from clinical trials' treatment information and match their names with records on the DrugBank database (20).

4) We also collected clinical trials data at the experimental readout time 6/15/2020 (C615 dataset) (Dataset 11).

Note that some methods do not provide prediction for every drug in the full dataset. While that would make a fair comparison of the methods challenging, we note that ground-truth information described above is available for drugs predicted by all pipelines (except for P3, hence it is harder to compare this pipeline with the other 11). Finally, we note that we adopted a conservative approach by evaluating predictive performance using the rankings across all 6,340 drugs, not only 918 experimentally screened drugs. For example, it is possible to conceive that a particular topmost prediction in a pipeline represents a positive drug; however, that is impossible to know if the predicted drug was not included in experimental screening. Because of that, the reported precision and recall values represent conservative estimates of prediction performance, i.e., the values are lower than what one could obtain if the analysis was limited to only experimentally screened drugs. To determine the significance of predictive power, we calculated the expected number of positive drugs among top-K drugs for each pipeline and compared the expected values with the observed precision and recall values. To this end, we calculated the expected number of positive drugs by taking into account (a) the number of drugs for which ground-truth information is available, and (b) the number of drugs for which a pipeline makes predictions. We used an exact one-tailed binomial test (p-value < 0.05) to test whether a top-K list returned by a pipeline is biased towards containing more positive drugs than what we would expect on average by pure chance had the ranking be a random one.

6 Rank Aggregation Algorithms (RAAs)

Rank aggregation is concerned with how to combine several independently constructed rankings into one final ranking that represents a consensus ranking, i.e., a collective opinion of prediction methods that is representative of all rankings returned by the methods (51). The classical consideration for specifying the final ranking is to maximize the number of pairwise agreements between the final ranking and each input ranking. Unfortunately, this objective, known as the Kemeny consensus, is NP-hard to compute (51, 52), which has motivated the development of methods that either use heuristics or approximate the Kemeny optimal ranking (51, 53–55).

6.1 Average Rank Method

The Average Rank method follows the most straightforward way to integrate multiple rankings. For each drug, it calculates a simple rank average over 12 rankings returned by the pipelines to obtain the overall

ranking. While the Average Rank method is a popular ad-hoc rank aggregation strategy, many studies (56–58), including ours, found that studying the average ranks can be a poor aggregation approach. Next, we briefly overview methods that realize more sophisticated approaches to obtain the overall ranking.

6.2 Borda Method

The Borda method (59) is one of most commonly used rank aggregation methods. Briefly, the method proceeds as follows. Given are k rankings exist, $R_1, R_2, \ldots, R_k$. For each drug $a \in R_i$, a is assigned a score $B_i(a)$ equal to the number of drugs that a outranks in ranking R_i. The Borda count $B(a)$ of drug a is then calculated as $\sum_{i=1}^{k} B_i(a)$. Finally, drugs are sorted in the descending order based on their Borda counts to create a consensus ranking. Theoretically, Borda method offers a guarantee on approximating Kemeny consensus. In particular, Borda method is a 5-approximation algorithm of the Kemeny optimal ranking (54). We used the Python package rankaggregation for computing the Borda ranking.

6.3 Dowdall Method

The Dowdall method (60) is a modified form of the Borda method that has been widely used in political elections in many countries. Intuitively, individual pipelines make predictions for drugs, which are interpreted as preferences of the pipeline. For a pipeline, its 1st choice gets a score of 1, its 2nd choice gets 1/2, its 3rd choice gets 1/3, and so on. Drug with the largest total score across pipelines wins. Formally, let be given k rankings, $R_1, R_2, \ldots, R_k$. For each drug $a \in R_i$, a is first assigned a score $D_i(a)$ equal to the reciprocal of drug's rank in ranking R_i. The total score $D(a)$ is then calculated as $\sum_{i=1}^{k} D_i(a)$. Candidates are sorted in descending order based on their total score to create a consensus ranking. We used the Python package rankaggregation for computing the Dowdall ranking.

6.4 CRank

The CRank algorithm (61) starts with ranked lists of drugs, R_r, each one arising from a different pipeline, *r*. Each ranked list is partitioned into equally sized groups, called bags. Each bag i in ranked list R_r has attached importance weight K_r^i whose initial values are all equal. CRank uses a two-stage iterative procedure to aggregate the individual rankings by taking into account uncertainty that is present across ranked lists. After initializing the aggregate ranking R as a weighted average of ranked lists R_r, CRank alternates between the following two stages until no changes were observed in the aggregated ranking R. (1) First, it uses the current aggregated ranking R to update the importance weights K_r^i for each ranked list. For that purpose, the top-ranked drugs in R serve as a temporary gold standard. Given bag i and ranked list R_r, CRank updates importance weight K_r^i based on how many drugs from the temporary gold standard appear in bag i using Bayes factors (62, 63). (2) Second, the ranked lists are re-aggregated based on the importance weights calculated in the previous stage. The updated importance weights are used to revise

R in which the new rank $R(a)$ of drug a is expressed as: $R(a) = \sum_r K_r^{i_r(a)} R_r(a)$, where $K_r^{i_r(a)}$ indicates the importance weight of bag $i_r(a)$ of drug a for ranking r, and $R_r(a)$ is the rank of a according to r. By using an iterative approach, CRank allows for the importance of a ranked list returned by an individual pipeline not to be predetermined, i.e., a-priori fixed, and to vary across drugs. The final output is a global ranked list R of drugs that represents the collective opinion of all drug repurposing prediction algorithms. In all experiments, we set the number of bags to 1,000, the size of the temporary gold standard to 0.5% of the total number of drugs in R, and the maximum number of iterations to 50. In all cases, the algorithm converged, in fewer than 20 iterations (62, 63). The pipelines' ranked lists and CRank's aggregation are provided in Dataset 12. The Python source code implementation of CRank is available at https://github.com/mims-harvard/crank (raa.py).

6.5 Comparison of RAAs.

What explains CRank's outstanding performance across all datasets? Each RAA aims to approximate the optimal Kemeny consensus, which offers the best agreement with all 12 prediction pipelines. As this consensus remains unknown (NP-hard), we cannot assess how well the different RAA methods approximate it. We do, however, have a ground-truth ranking, offered by the experimental and clinical datasets (E918 and CT415). We assigned rank 1 to the strong drugs, rank 2 to the weak drugs, and rank 3 to the no-effect drugs, allowing us to measure the Kemeny score for each aggregated list, representing the fraction of pairwise disagreements between the respective ranked list and the experimental outcomes. For $\mathrm{K} = 100$, the Kemeny score of the Average Rank method is infinite for E918, as there are no positive drugs among the top 100. In contrast, for the Borda count, we obtain a Kemeny score of KS = 0.7131, indicating that 71% of all drug pairs in the ranked list of Borda method disagrees with the ground-truth ranking in the E918 dataset. Note that the theoretical expectation for a purely random ranking is KS = 0.5, meaning that 50% of all drug pairs in the random reference are flipped, i.e., while with KS = 0.4545 Dowdall does better than random, we observe a much lower KS = 0.2679 for CRank. We measured the Kemeny score for multiple values, for both datasets (E918 and CT415), finding that for K<250 (top drugs), CRank offers the best agreement with the outcomes.

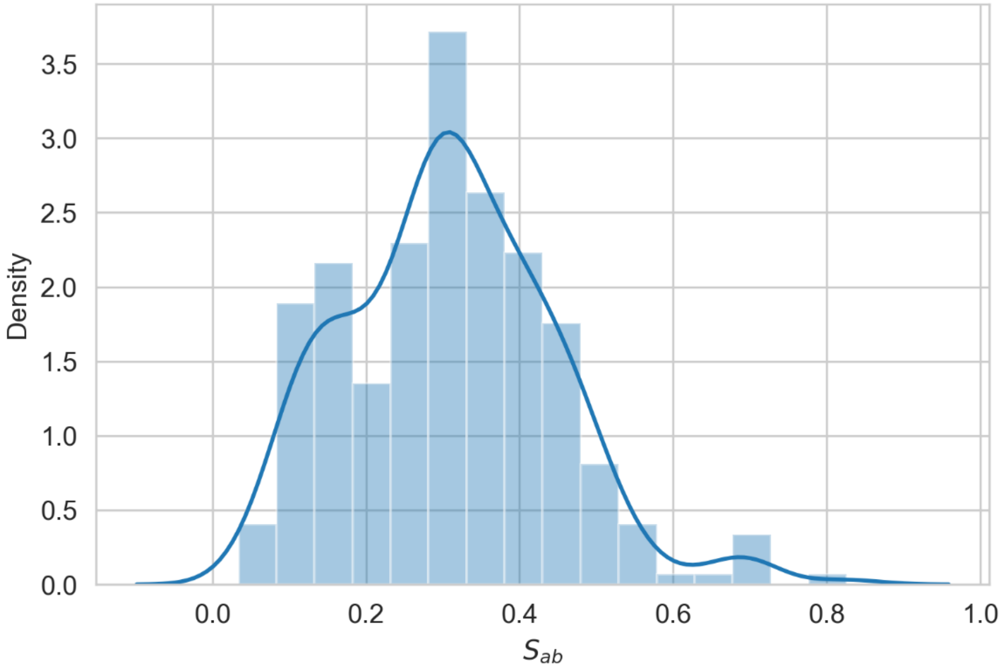

Fig. S1. Distribution of the Network Overlap Measure S_{vb} Between 299 Diseases and COVID-19 Targets. S_{vb} values represent the network-based overlap between SARS-COV2 targets v and the genes associated with each disease b.

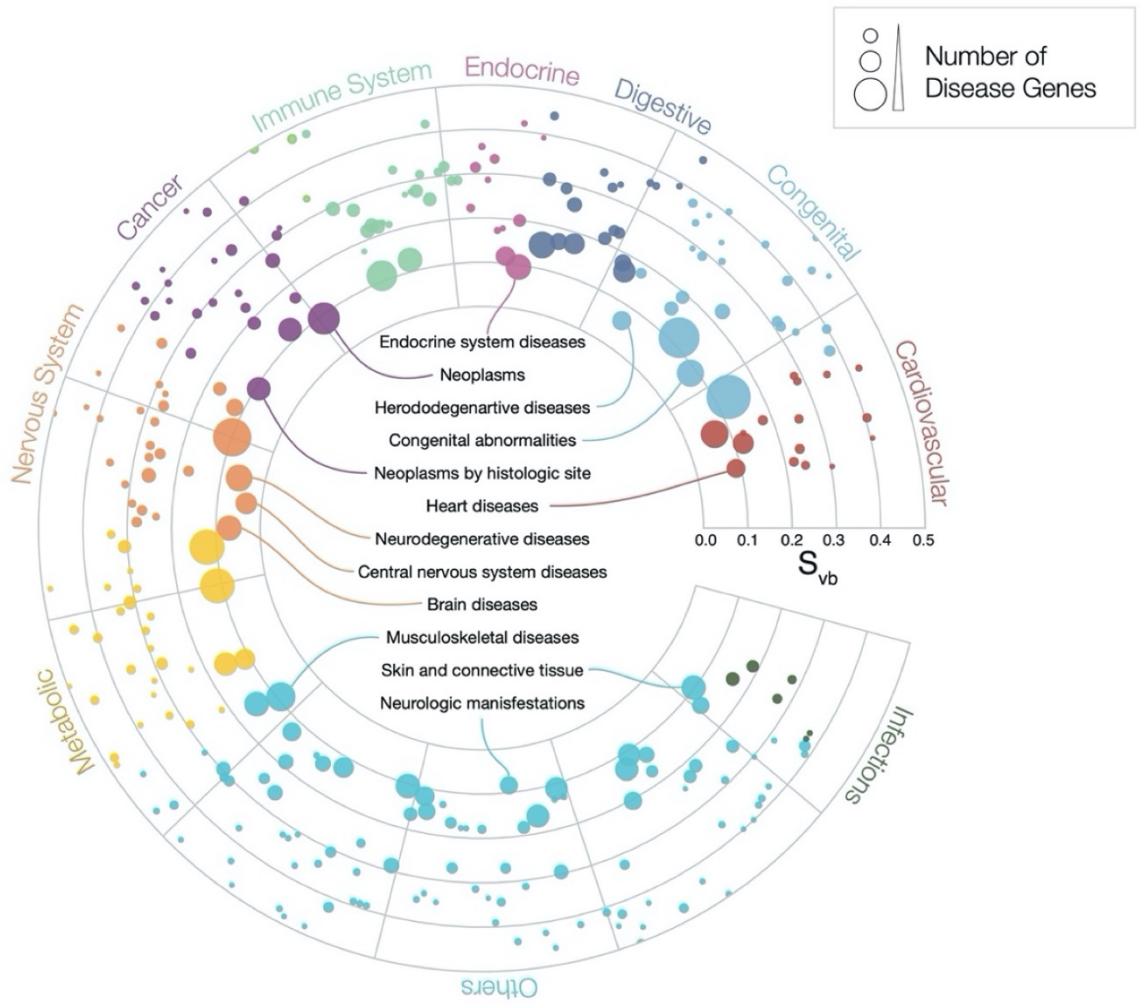

Fig. S2. Disease Comorbidity Measured by the Network Overlap Between COVID-19 Targets and 299 Diseases. The figure represents each disease as a circle whose radius reflects the number of disease genes associated with it (28). The diseases closest to the center, whose names are marked, are expected to have higher comorbidity with the COVID-19 outcome. The farther a disease is from the center, the more distant are its disease proteins from the COVID-19 viral targets. Disease Comorbidity. We measured the network proximity between COVID-19 targets and 299 diseases. The figure represents each disease as a circle whose radius reflects the number of disease genes associated with it (28). The diseases closest to the center, whose names are marked, are expected to have higher comorbidity with the COVID-19 outcome. The farther a disease is from the center, the more distant are its disease proteins from the COVID-19 viral targets.

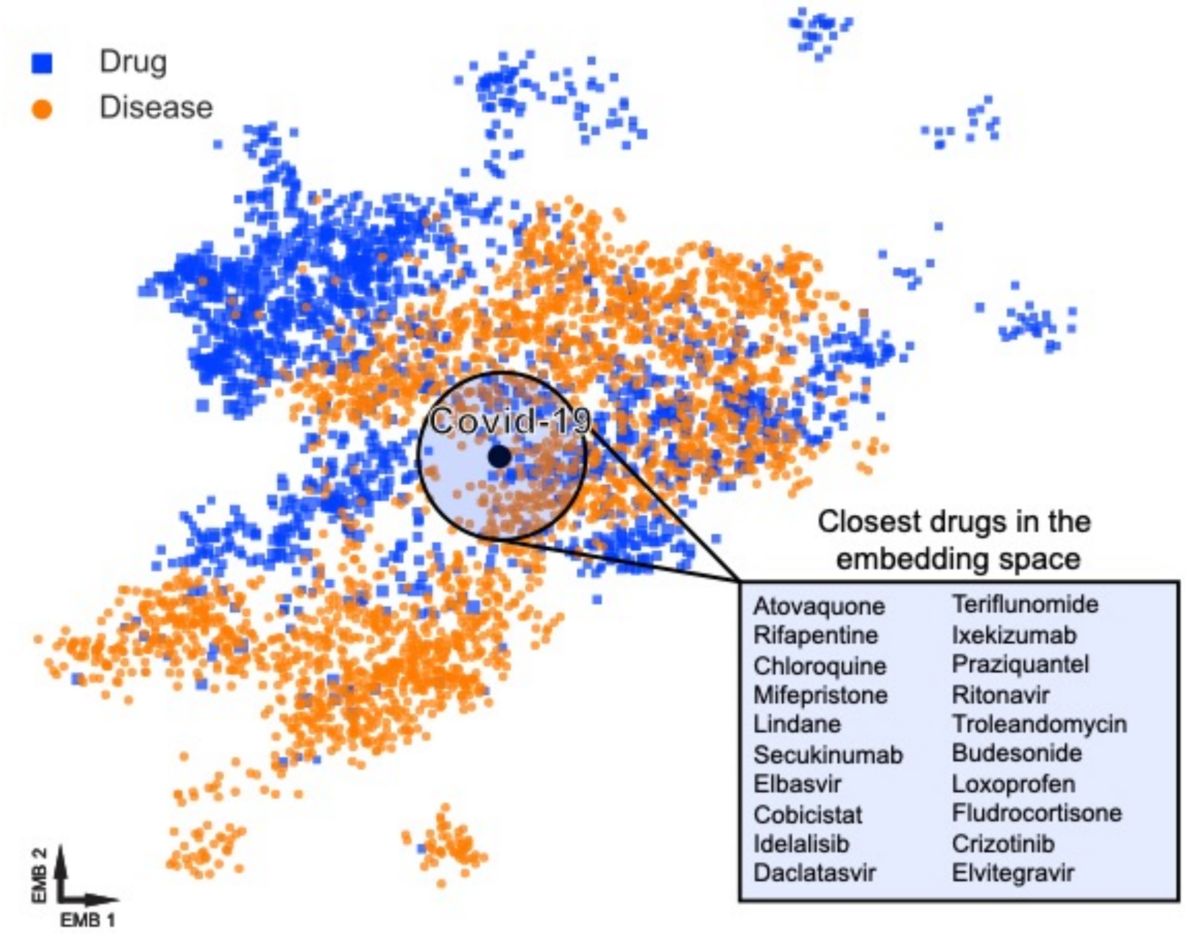

Fig. S3. Visualization of Drug and Disease Embeddings in AI-based Strategy for Drug Repurposing. Visualization of the learned embedding space. Every point represents a drug (in blue) or a disease (in orange). If a drug and a disease are embedded close together in this space, this means the local interaction neighborhoods of the drug and the disease in the multimodal graph are predictive of whether the drug can treat the disease.

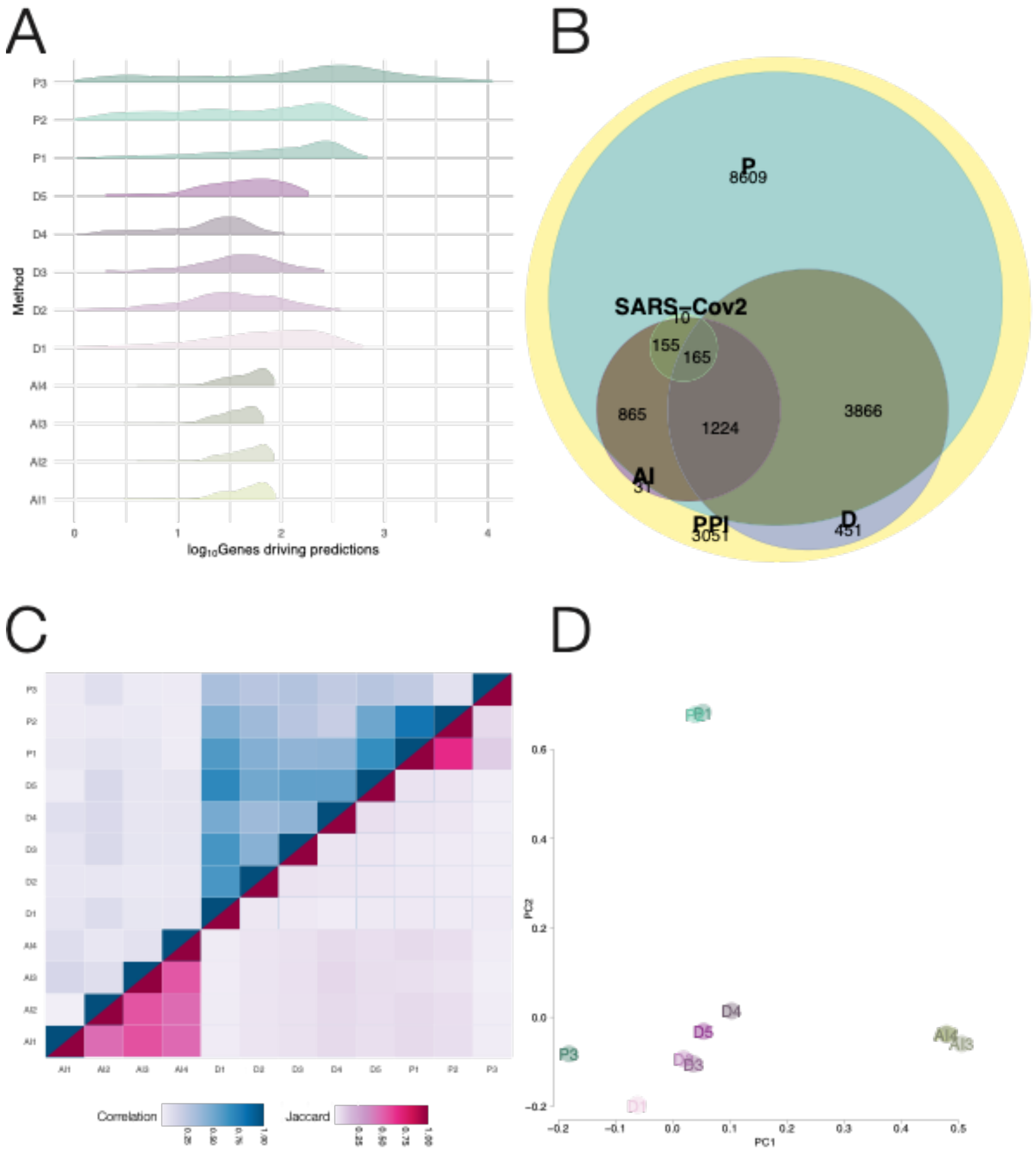

Fig. S4. Similarities and Differences of the Explanatory Subgraphs. (A) Distribution of the size of the subnetworks predicted varies according to the method. The AI methods have a smaller variance in the size, while methods based on proximity tend to have higher variances. (B) Gene overlap of the methods involved with subgraphs for each method. Proximity and Diffusion based methods explore the PPI in a much vast and diverse way than the AI methods (C) Methods inside the same pipeline tend to select similar genes, the similarity of selected genes across methods is different (Jaccard Index), those genes, interestingly, also do not lie in similar neighborhood (similarity), meaning that not only do the genes not overlap across methods, but the vicinity the methods explore are also different. (D) Another measure used to understand methods similarity involved using the PCA of gene drug pairs, showing that AI methods are fairly consistent in what they observe, and similarly, P1 and P2. Diffusion methods have a higher variance in gene-drug pair predictions and have a larger spread of their module; as expected, P3 is far from other proximity measures.

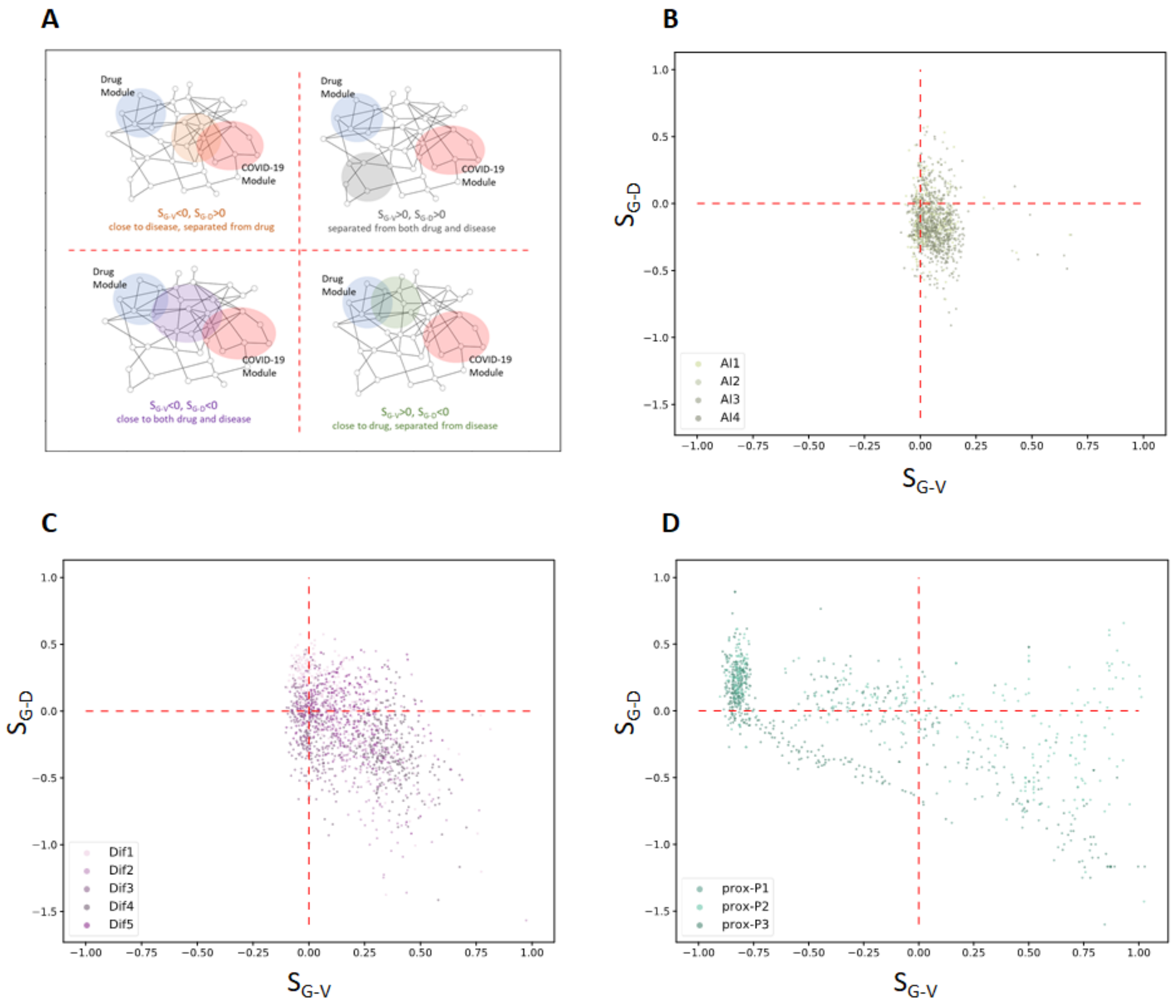

Fig. S5. The Separation Plot for 320 Drugs. For each drug, we identified the predictive subgraph for each predictive pipeline. For each subgraph G, we compute the separation between the subgraph G and drug targets as $S_{G\text{-}D}$ and separation between the subgraph and SARS-CoV-2 targets as $S_{G\text{-}V}$. We plot each subgraph as a dot with the two separation values as coordinates to form the plot above. (A) a schematic showing the network pattern represented by each quadrant; (B)- (D): plot for subgraphs in AI, Diffusion, Proximity pipelines, respectively. Each method's subgraphs locate in different regions in the plot, suggesting that they use complimentary regions of the PPI to make predictions.

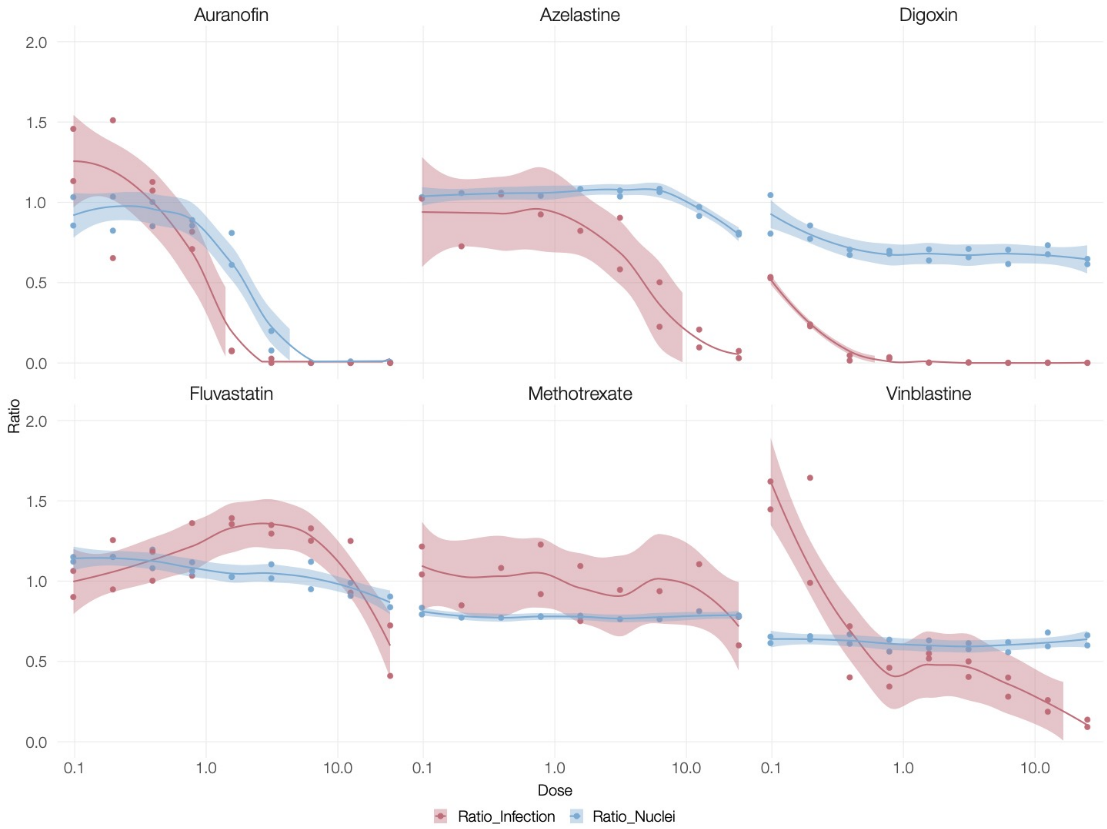

Fig. S6. Drug Response Curves for the S&W drugs validated in human cell line. From the top 200 drugs, we re-screened the ones that showed viral load deduction in the first screening using Huh7 cells, that were challenged in vitro with SARS-CoV-2 virus and treated with the drug at a nine-point dilution series from 25μM to 100nM. The result shows that Auranofin, Azelastine, Vinblastine, Fluvastatin, Methotrexate and Digoxin are able to reduce the viral load with particularly strong effect observed for auranofin. Note that, methodextrate is effective only in the last dose, and therefore, still classified as effective, while the other drugs can be effective in multiple dose points. The ratio nuclei is the ratio of nuclei count in the treated cells, normalized by the nuclei count in the controls; the ratio infection is the infected cell ratio normalized by the nuclei count in control.

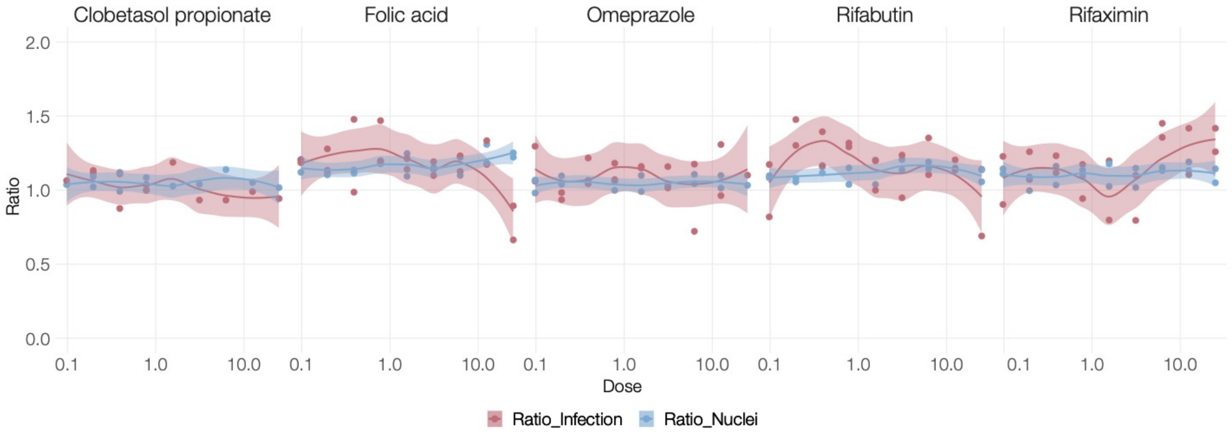

Fig. S7. Drug Response Curves for the non-effect drugs validated in human cell line. By re-screening the drugs that showed viral load deduction from top 200 ranked ones in the first screening using Huh7 cells, we are able to capture that five (out 11 drugs) did not show significant reduction on the viral load. The ratio nuclei is the ratio of nuclei count in the treated cells, normalized by the nuclei count in the controls; the ratio infection is the infected cell ratio normalized by the nuclei count in control.

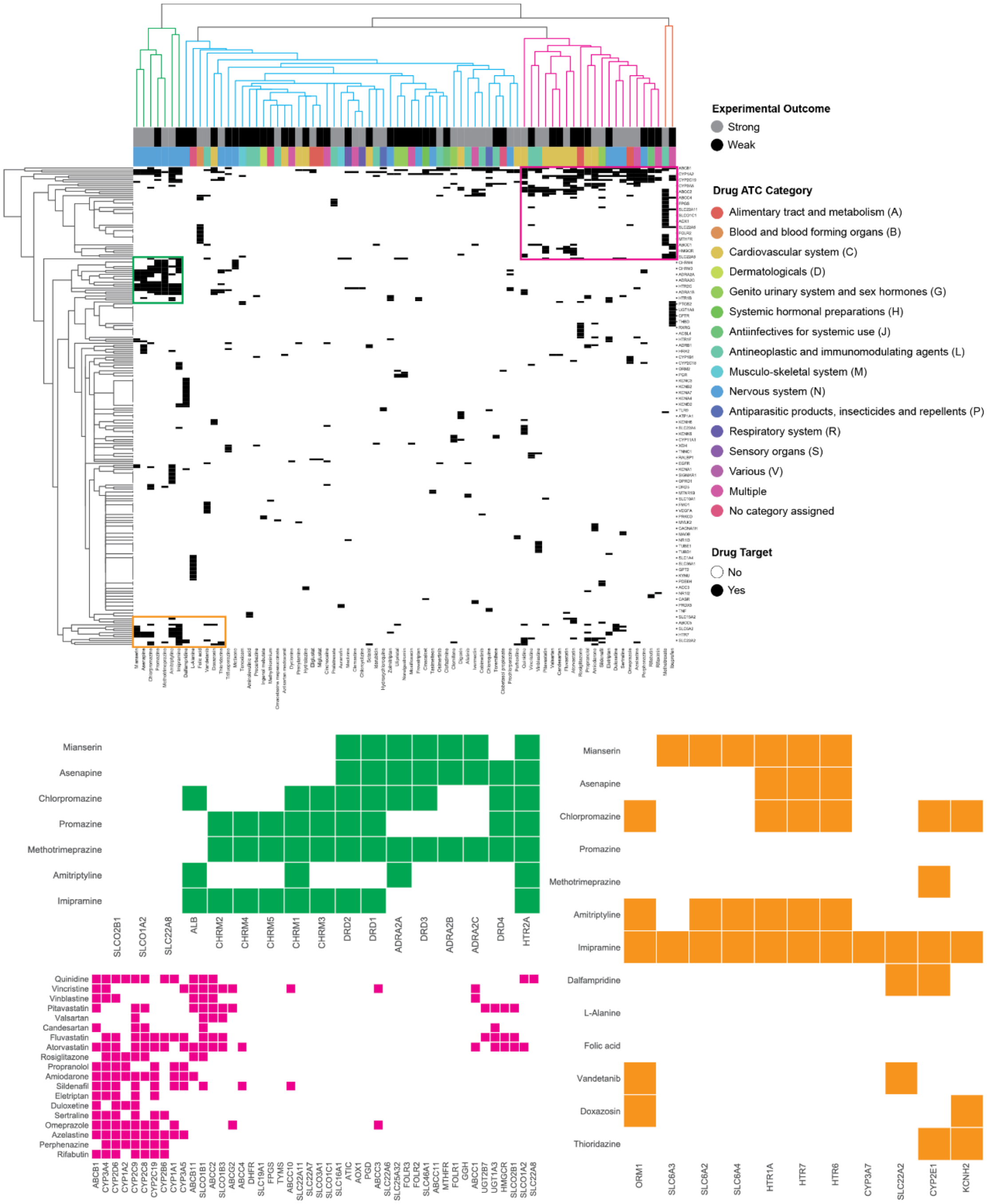

Fig. S8. Hierarchical Clustering Highlights Groups of Drugs with Similar Target Profiles. Heatmap showing 77 S&W drugs from the E918 dataset and their respective targets (colored cells). Clustering performed using Euclidean distance and single linkage.

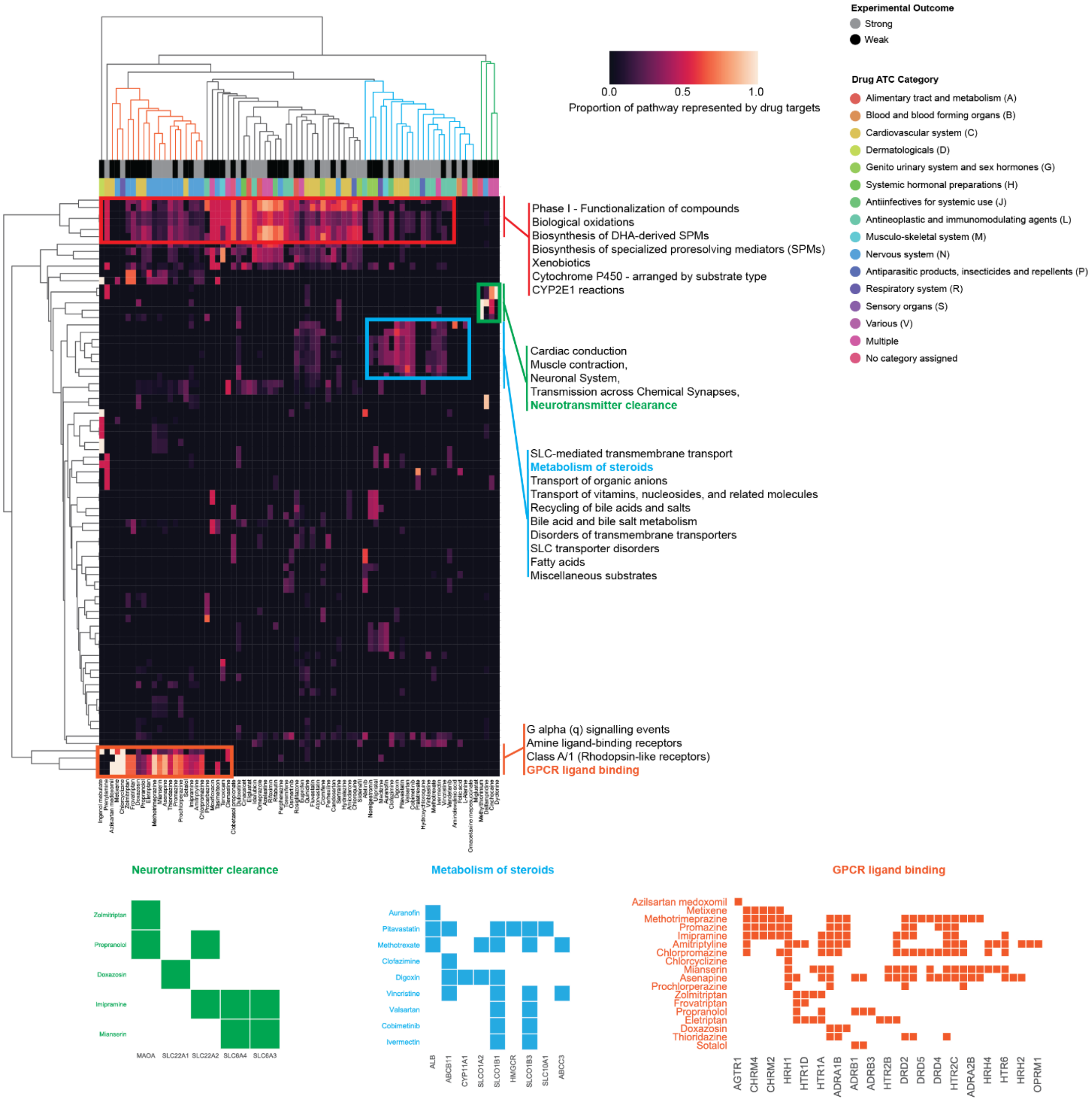

Fig. S9. Pathway Enrichment. Heatmap showing successful (S&W) drugs in the E918 dataset and their respective Reactome pathways in which their targets are enriched. Hierarchical clustering (Euclidean, single linkage) highlights different groups of drugs with similar pathway profiles. We highlight the pathways for three drug clusters, emphasizing the proteins targeted in one exemplary pathway for each cluster.

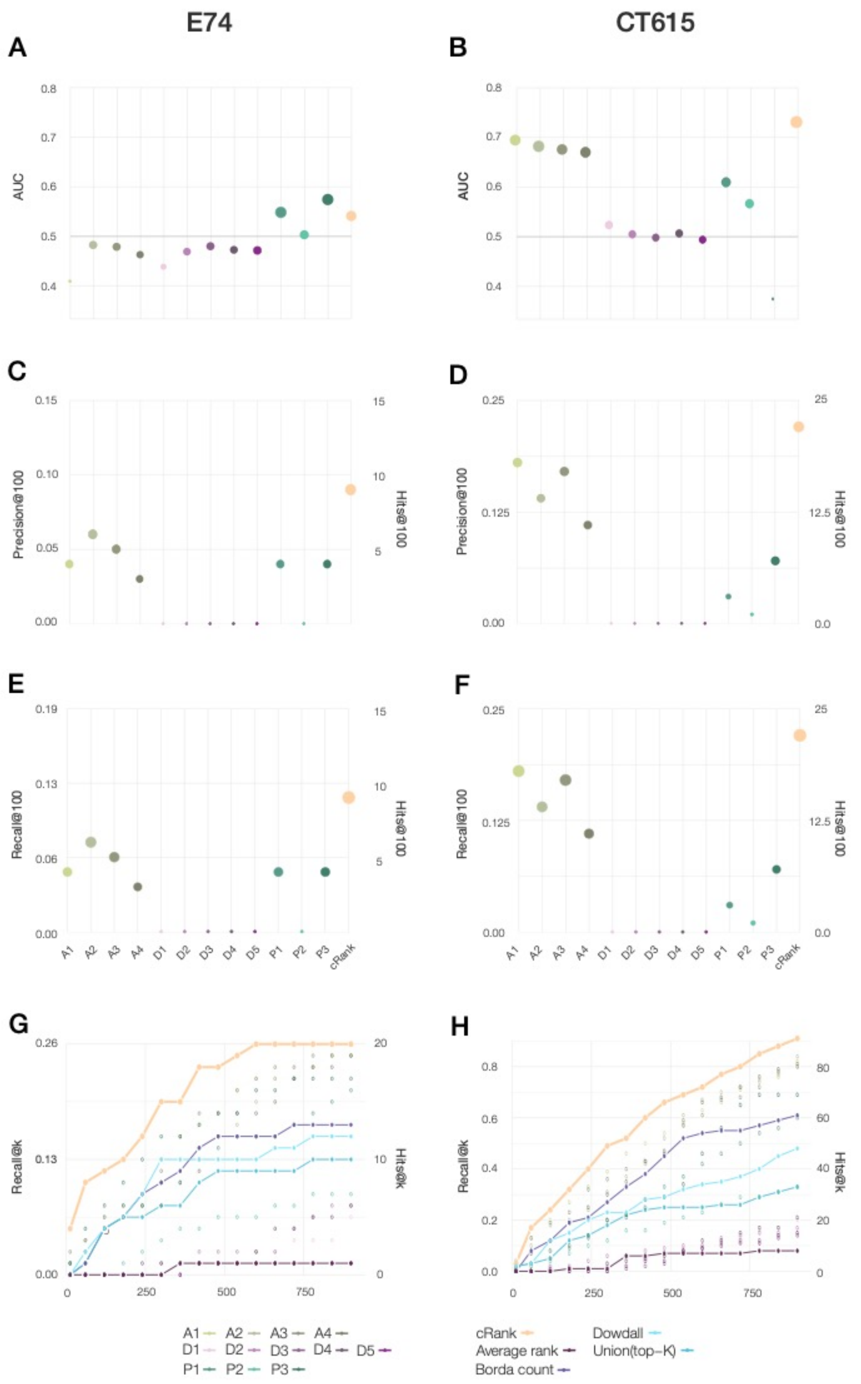

Fig. S10. Performance of the Different Predictive Pipelines. (A,B) AUC (Area under the Curve), (C,D) precision at 100, and (E,F) recall, for twelve pipelines tested for drug repurposing, using as a gold standard the S&W drugs in E74 (left panel, experimentally validated dataset from expert curation and drug selection) and CT615 (right panel, drugs in clinical trials until July 15th 2020). (G,H) The top precision and recall for the different rank aggregation methods (connected points), compared to the individual pipelines (empty symbols) documenting the strong predictive performance of CRank. CT06 presents, in most cases, higher hit rates, precision and recalls when compared to E74.

Table S1. Performance evaluation using the E918 dataset as ground-truth (Fig 4C,E)

	AUC (Only Strong)	AUC	Precision@100	Recall@100
CRank	0.65	0.53	9% (0.750%, p=7.01 x 10^{-8})	11.7% (0.9%, p=2.61 x 10^{-8})
P1	0.68	0.53	7% (0.467%, p=3.77 x 10^{-7})	9.1% (0.6%, p=2.52 x 10^{-7})
P2	0.57	0.46	0% (0.458%, p=1.00)	0% (0.5%, p=1.00)
P3	0.58	0.49	5% (2.453%, p=0.090)	6.5% (2.3%, p= 0.031)
D1	0.56	0.53	2% (0.062%, p=0.001)	2.6% (0.1%, p=9.32 x 10^{-4})
D2	0.59	0.53	2% (0.075%, p=0.002)	2.6% (0.1%, p=2.29 x 10^{-3})
D3	0.49	0.51	1% (0.062%, p=0.061)	1.3% (0.1%, p=0.061)
D4	0.59	0.53	1% (0.037%, p=0.037)	1.3% (0.1%, p=0.037)
D5	0.49	0.51	1% (0.062%, p=0.061)	1.3% (0.1%, p=0.061)
AI1	0.50	0.50	4% (2.779%, p=0.302)	5.2% (3.3%, p=0.024)
AI2	0.59	0.52	9% (2.922%, p=0.002)	11.7% (3.7%, p=2.14 x 10^{-3})
AI3	0.50	0.50	6% (2.635%, p=0.047)	7.8% (3.2%, p=0.037)
AI4	0.59	0.52	6% (2.922%, p=0.049)	7.8% (3.5%, p=0.054)

The values in the brackets represent expected values, i.e. expected Recall@100 or expected Precision@100, followed by p-values – binomial test.

Table S2. Performance evaluation using the CT415 dataset (Fig 4D,F)

	AUC	Precision@100	Recall@100
CRank	0.78	12% (0.360%, $p=3.06 \times 10^{-15}$)	32.4% (0.5%, $p=2.17 \times 10^{-19}$)
P1	0.58	1% (0.224%, p=0.201)	2.7% (0.6%, p=0.202)
P2	0.56	0% (0.220%, p=1.00)	0.0% (0.6%, p=1.00)
P3	0.42	2% (1.790%, p=0.538)	5.4% (2.6%, p=0.245)
D1	0.55	0% (0.030%, p=1.0)	0% (0.1%, p=1.00)
D2	0.53	0% (0.036%, p=1.0)	0% (0.1%, p=1.00)
D3	0.53	0% (0.030%, p=1.0)	0% (0.1%, p=1.00)
D4	0.52	0% (0.018%, p=1.0)	0% (0.1%, p=1.00)
D5	0.52	0% (0.302%, p=1.0)	0% (0.1%, p=1.00)
AI1	0.73	12% (1.335%, $p=7.39 \times 10^{-9}$)	32.4% (2.3%, $p=2.41 \times 10^{-11}$)
AI2	0.76	10% (1.404%, $p=1.29 \times 10^{-6}$)	27.0% (2.3%, $p=8.25 \times 10^{-9}$)
AI3	0.72	11% (1.267%, $p=4.52x \times 10^{-8}$)	29.7% (2.3%, $p=4.74 \times 10^{-10}$)
AI4	0.76	9% (1.404%, $p=1.06 \times 10^{-5}$)	24.3% (2.3%, $p=1.26 \times 10^{-7}$)

The values in the brackets represent expected values, i.e. expected Recall@100 or expected Precision@100, followed by p-values – binomial test.

Dataset S1. SARS-COV2-Human Interactome. Protein-protein interactions between 29 SARS-COV2 proteins and 332 human proteins detected by affinity purification followed by mass spectrometry (dataset retrieved from Gordon et al (2020)).

Dataset S2. Protein-Protein Human Interactome. 332,749 pairwise binding interactions between 18,508 human proteins.

Dataset S3. List of drugs and their respective targets. Retrieved from the DrugBank database.

Dataset S4. List of 17,222 differentially expressed genes identified by exposure of 793 drugs in different cell lines. Data obtained from the DrugBank database. Differential Expressed Genes used for P3.

Dataset S5. Network Overlap Between 299 Diseases and SARS-COV2 Targets. The S_{vb} measure captures the network-based overlap between SARS-COV2 targets v and the gene pool associated with disease b.

Dataset S6. Embedding vectors. Representations of diseases as learned by the GNN model. Each row in the file contains the embedding vector for a particular disease.

Dataset S7. Embedding vectors. Representations of drugs as learned by the GNN model. Each row in the file contains the embedding vector for a particular drug.

Dataset S8. The E918 dataset. List of 918 drugs screened for their efficacy in inhibiting SARS-CoV-2 in VeroE6 cells and their experimental outcome.

Dataset S9. The E74 dataset. Experimental outcomes of 74 compounds selected by a medical doctor among the top 10% of all drug predictions that were available for purchase.

Dataset S10. Drugs Under Evaluation in Clinical Trials for Treating COVID-19 (as of April 2020) (C415 dataset).

Dataset S11. Drugs Under Evaluation in Clinical Trials for Treating COVID-19 (as of June 2020) (C615 dataset).

Dataset S12. Drug Rankings. Ranking of each drug obtained by the 12 pipelines and their aggregation with CRank.

References:

1. K. Luck, *et al.*, A reference map of the human binary protein interactome. *Nature* **580**, 402–408 (2020).

2. R. Mosca, A. Céol, P. Aloy, Interactome3D: adding structural details to protein networks. *Nature methods* **10**, 47–53 (2013).

3. M. J. Meyer, J. Das, X. Wang, H. Yu, INstruct: a database of high-quality 3D structurally resolved protein interactome networks. *Bioinformatics (Oxford, England)* **29**, 1577–9 (2013).

4. M. J. Meyer, *et al.*, Interactome INSIDER: a structural interactome browser for genomic studies. *Nature methods* **15**, 107–114 (2018).

5. M. J. Cowley, *et al.*, PINA v2.0: mining interactome modules. *Nucleic acids research* **40**, D862-5 (2012).

6. L. Licata, *et al.*, MINT, the molecular interaction database: 2012 update. *Nucleic Acids Research* **40**, D857–D861 (2012).

7. A. Chatr-Aryamontri, *et al.*, The BioGRID interaction database: 2017 update. *Nucleic acids research* **45**, D369–D379 (2017).

8. J. Das, H. Yu, HINT: High-quality protein interactomes and their applications in understanding human disease. *BMC Systems Biology* **6** (2012).

9. G. Alanis-Lobato, M. A. Andrade-Navarro, M. H. Schaefer, HIPPIE v2.0: enhancing meaningfulness and reliability of protein-protein interaction networks. *Nucleic acids research* **45**, D408–D414 (2017).

10. Di. Alonso-López, *et al.*, APID database: Redefining protein-protein interaction experimental evidences and binary interactomes. *Database* **2019**, 1–8 (2019).

11. T. Li, *et al.*, A scored human protein-protein interaction network to catalyze genomic interpretation. *Nature Methods* **14**, 61–64 (2016).

12. E. L. Huttlin, *et al.*, Architecture of the human interactome defines protein communities and disease networks. *Nature* **545**, 505–509 (2017).

13. M. Y. Hein, *et al.*, A Human Interactome in Three Quantitative Dimensions Organized by Stoichiometries and Abundances. *Cell* **163**, 712–723 (2015).

14. C. Wan, *et al.*, Panorama of ancient metazoan macromolecular complexes. *Nature* **525**, 339–44 (2015).

15. F. Cheng, P. Jia, Q. Wang, Z. Zhao, Quantitative network mapping of the human kinome interactome reveals new clues for rational kinase inhibitor discovery and individualized cancer therapy. *Oncotarget* **5**, 3697–710 (2014).

16. P. V Hornbeck, *et al.*, PhosphoSitePlus, 2014: mutations, PTMs and recalibrations. *Nucleic acids research* **43**, D512-20 (2015).

17. D. Fazekas, *et al.*, SignaLink 2 - a signaling pathway resource with multi-layered regulatory

networks. *BMC systems biology* **7**, 7 (2013).

18. K. Breuer, *et al.*, InnateDB: systems biology of innate immunity and beyond--recent updates and continuing curation. *Nucleic acids research* **41**, D1228-33 (2013).

19. D. E. Gordon, *et al.*, A SARS-CoV-2-Human Protein-Protein Interaction Map Reveals Drug Targets and Potential Drug-Repurposing. *bioRxiv*, 2020.03.22.002386 (2020).

20. D. S. Wishart, *et al.*, DrugBank 5.0: a major update to the DrugBank database for 2018. *Nucleic acids research* **46**, D1074–D1082 (2018).

21. E. Guney, J. Menche, M. Vidal, A.-L. L. Barábasi, Network-based in silico drug efficacy screening. *Nature Communications* **7**, 10331 (2016).

22. J. Lonsdale, *et al.*, The Genotype-Tissue Expression (GTEx) project. *Nature Genetics* **45**, 580–585 (2013).

23. G. Grasselli, *et al.*, Baseline Characteristics and Outcomes of 1591 Patients Infected With SARS-CoV-2 Admitted to ICUs of the Lombardy Region, Italy. *JAMA* (2020) https:/doi.org/10.1001/jama.2020.5394.

24. N. Gulbahce, *et al.*, Viral perturbations of host networks reflect disease etiology. *PLoS Computational Biology* **8**, 1002531 (2012).

25. J. Park, D. S. Lee, N. A. Christakis, A. L. Barabási, The impact of cellular networks on disease comorbidity. *Molecular Systems Biology* **5**, 262 (2009).

26. C. A. Hidalgo, N. Blumm, A. L. Barabási, N. A. Christakis, A Dynamic Network Approach for the Study of Human Phenotypes. *PLoS Computational Biology* **5** (2009).

27. D. S. Lee, *et al.*, The implications of human metabolic network topology for disease comorbidity. *Proceedings of the National Academy of Sciences of the United States of America* **105**, 9880–9885 (2008).

28. J. Menche, *et al.*, Disease networks. Uncovering disease-disease relationships through the incomplete interactome. *Science (New York, N.Y.)* **347**, 1257601 (2015).

29. N. Chen, *et al.*, Epidemiological and clinical characteristics of 99 cases of 2019 novel coronavirus pneumonia in Wuhan, China: a descriptive study. *The Lancet* **395**, 507–513 (2020).

30. D. Wang, *et al.*, Clinical Characteristics of 138 Hospitalized Patients with 2019 Novel Coronavirus-Infected Pneumonia in Wuhan, China. *JAMA - Journal of the American Medical Association* (2020) https:/doi.org/10.1001/jama.2020.1585.

31. C. Huang, *et al.*, Clinical features of patients infected with 2019 novel coronavirus in Wuhan, China. *The Lancet* **395**, 497–506 (2020).

32. A. Giacomelli, *et al.*, Self-reported olfactory and taste disorders in SARS-CoV-2 patients: a cross-sectional study. *Clinical Infectious Diseases* (2020) https:/doi.org/10.1093/cid/ciaa330 (April 2, 2020).

33. M. Zitnik, M. Agrawal, J. Leskovec, Modeling polypharmacy side effects with graph convolutional

networks. *Bioinformatics* **34**, i457–i466 (2018).

34. J. Gilmer, S. S. Schoenholz, P. F. Riley, O. Vinyals, G. E. Dahl, Neural message passing for quantum chemistry in *ICML*, (2017), pp. 1263–1272.

35. P. Veličković, *et al.*, Graph attention networks. *ICLR* (2018).

36. D. Kingma, J. Ba, Adam: A method for stochastic optimization. *arXiv:1412.6980* (2014).

37. X. Glorot, Y. Bengio, Understanding the difficulty of training deep feedforward neural networks in *AISTATS*, (2010), pp. 249–256.

38. W. Hamilton, P. Bajaj, M. Zitnik, D. Jurafsky, J. Leskovec, Embedding logical queries on knowledge graphs in *NIPS*, (2018), pp. 2026–2037.

39. N. Srivastava, others, Dropout: a simple way to prevent neural networks from overfitting. *JMLR* **15**, 1929–1958 (2014).

40. W. Hamilton, Z. Ying, J. Leskovec, Inductive representation learning on large graphs in *NIPS*, (2017), pp. 1024–1034.

41. E. Becht, *et al.*, Dimensionality reduction for visualizing single-cell data using {UMAP}. *Nature Biotechnology* **37**, 38 (2019).

42. M. Cao, *et al.*, Going the Distance for Protein Function Prediction: A New Distance Metric for Protein Interaction Networks. *PLoS ONE* (2013) https:/doi.org/10.1371/journal.pone.0076339.

43. C. C. Aggarwal, A. Hinneburg, D. A. Keim, On the Surprising Behavior of Distance Metrics in High Dimensional Space in *Database Theory --- ICDT 2001*, J. den Bussche, V. Vianu, Eds. (Springer Berlin Heidelberg, 2001), pp. 420–434.

44. R. Ying, D. Bourgeois, J. You, M. Zitnik, J. Leskovec, GNNExplainer: Generating Explanations for Graph Neural Networks (2019).

45. C. Ritz, F. Baty, J. C. Streibig, D. Gerhard, Dose-Response Analysis Using R. *PLOS ONE* **10**, e0146021 (2015).

46. C. Ritz, J. C. Streibig, Bioassay analysis using R. *Journal of Statistical Software* **12**, 1–22 (2005).

47. M. Honma, G. Tamura, K. Shirato, T. Takishima, Effect of an oral gold compound, auranofin, on non-specific bronchial hyperresponsiveness in mild asthma. *Thorax* (1994) https:/doi.org/10.1136/thx.49.7.649.

48. H. A. Rothan, *et al.*, The FDA-approved gold drug auranofin inhibits novel coronavirus (SARS-COV-2) replication and attenuates inflammation in human cells. *Virology* (2020) https:/doi.org/10.1016/j.virol.2020.05.002.

49. T. Sing, O. Sander, N. Beerenwinkel, T. Lengauer, ROCR: Visualizing classifier performance in R. *Bioinformatics* (2005) https:/doi.org/10.1093/bioinformatics/bti623.

50. H. Wickham, ggplot2. *Wiley Interdisciplinary Reviews: Computational Statistics* (2011) https:/doi.org/10.1002/wics.147.

51. C. Dwork, R. Kumar, M. Naor, D. Sivakumar, Rank aggregation methods for the web in *Proceedings of the 10th International Conference on World Wide Web*, (2001), pp. 613–622.

52. J. Bartholdi, C. A. Tovey, M. A. Trick, Voting schemes for which it can be difficult to tell who won the election. *Social Choice and welfare* **6**, 157–165 (1989).

53. F. Schalekamp, A. van Zuylen, Rank Aggregation: Together We are Strong. *Proc of 11th ALENEX* **18**, 38–51 (2009).

54. D. Coppersmith, L. Fleischer, A. Rudra, Ordering by weighted number of wins gives a good ranking for weighted tournaments. *Proceedings of the Annual ACM-SIAM Symposium on Discrete Algorithms*, 776–782 (2006).

55. N. Ailon, M. Charikar, A. Newman, Aggregating inconsistent information: Ranking and clustering. *Proceedings of the Annual ACM Symposium on Theory of Computing*, 684–693 (2005).

56. K. Eilbeck, A. Quinlan, M. Yandell, Settling the score: variant prioritization and Mendelian disease. *Nature Publishing Group* (2017) https:/doi.org/10.1038/nrg.2017.52.

57. O. Zolotareva, M. Kleine, A Survey of Gene Prioritization Tools for Mendelian and Complex Human Diseases. *Journal of Integrative Bioinformatics* **16** (2019).

58. D. Guala, E. L. L. Sonnhammer, A large-scale benchmark of gene prioritization methods. *Scientific Reports* **7**, 1–10 (2017).

59. J. C. Borda, Memoire sur les elections au scrutin. *Mémoires de l'académie royale*, 657–664 (1781).

60. B. Reilly, Social choice in the south seas: Electoral innovation and the Borda count in the Pacific Island countries. *International Political Science Review* (2002) https:/doi.org/10.1177/0192512102023004002.

61. M. Zitnik, R. Sosic, J. Leskovec, Prioritizing Network Communities. *Nature Communications* **9**, 2544 (2018).

62. R. E. Kass, A. E. Raftery, Bayes factors. *Journal of the American Statistical Association* **90**, 773–795 (1995).

63. G. Casella, E. Moreno, Assessing robustness of intrinsic tests of independence in two-way contingency tables. *Journal of the American Statistical Association* **104**, 1261–1271 (2012).
